# Rapid and synchronous chemical induction of replicative‐like senescence via a small molecule inhibitor

**DOI:** 10.1111/acel.14083

**Published:** 2024-01-09

**Authors:** Spiros Palikyras, Konstantinos Sofiadis, Athanasia Stavropoulou, Adi Danieli‐Mackay, Vassiliki Varamogianni‐Mamatsi, David Hörl, Simona Nasiscionyte, Yajie Zhu, Ioanna Papadionysiou, Antonis Papadakis, Natasa Josipovic, Anne Zirkel, Aoife O'Connell, Gary Loughran, James Keane, Audrey Michel, Wolfgang Wagner, Andreas Beyer, Hartmann Harz, Heinrich Leonhardt, Grazvydas Lukinavicius, Christoforos Nikolaou, Argyris Papantonis

**Affiliations:** ^1^ Institute of Pathology University Medical Center Göttingen Göttingen Germany; ^2^ Institute for Bioinnovation Biomedical Sciences Research Center “Alexander Fleming” Vari Greece; ^3^ Clinical Research Unit 5002 University Medical Center Göttingen Göttingen Germany; ^4^ Faculty of Biology Ludwig Maximilians University Munich Munich Germany; ^5^ Cluster of Excellence on Cellular Stress Responses in Aging‐Associated Diseases (CECAD) University of Cologne Cologne Germany; ^6^ Center for Molecular Medicine Cologne University and University Hospital of Cologne Cologne Germany; ^7^ EIRNA Bio (formerly Ribomaps) Cork Ireland; ^8^ Helmholtz‐Institute for Biomedical Engineering RWTH Aachen University Medical School Aachen Germany; ^9^ Institute for Stem Cell Biology RWTH Aachen University Medical School Aachen Germany; ^10^ Department of NanoBiophotonics Max Planck Institute for Multidisciplinary Sciences Göttingen Germany; ^11^ Present address: Oncode Institute Hubrecht Institute‐KNAW and University Medical Center Utrecht Utrecht The Netherlands; ^12^ Present address: Single Cell Discoveries Utrecht The Netherlands

**Keywords:** 3D genome organization, cellular ageing, chromatin, SASP, senescence, single cell genomics

## Abstract

Cellular senescence is acknowledged as a key contributor to organismal ageing and late‐life disease. Though popular, the study of senescence in vitro can be complicated by the prolonged and asynchronous timing of cells committing to it and by its paracrine effects. To address these issues, we repurposed a small molecule inhibitor, inflachromene (ICM), to induce senescence to human primary cells. Within 6 days of treatment with ICM, senescence hallmarks, including the nuclear eviction of HMGB1 and ‐B2, are uniformly induced across IMR90 cell populations. By generating and comparing various high throughput datasets from ICM‐induced and replicative senescence, we uncovered a high similarity of the two states. Notably though, ICM suppresses the pro‐inflammatory secretome associated with senescence, thus alleviating most paracrine effects. In summary, ICM rapidly and synchronously induces a senescent‐like phenotype thereby allowing the study of its core regulatory program without confounding heterogeneity.

## Graphical abstract

We repurposed a small molecule inhibitor, inflachromene (ICM), to induce senescence to human primary cells. Within <6 days of ICM treatment, senescence hallmarks were uniformly induced across IMR90 cell populations, while SASP production was notably suppressed. Therefore, ICM will rapidly and synchronously induce a senescent‐like state in human cells without confounding heterogeneity from paracrine SASP signaling.

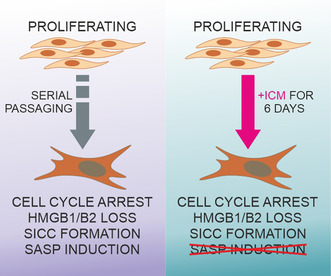

**Abbreviations**
*CCND2*
: cyclin D2 gene
*CDKN1A*
: cyclin‐dependent kinase inhibitor 1A gene
ChIP: chromatin immunoprecipitation
CTCF: CCCTC‐binding factor
DAPI: 4′,6‐diamidino‐2‐phenylindole
DNMT1: DNA methyltransferase 1
ECM: extracellular matrix
HDAC9: histone deacetylase 9
HMGA: high‐mobility group protein A
HMGB: high‐mobility group‐box protein
ICM: inflachromene
IMR90: human fetal lung fibroblasts
NFKB1: nuclear factor kappa B1
NFKBIA: nuclear factor kappa B inhibitor alpha
OIS: oncogene‐induced senescence
RELA: nuclear factor kappa B p65 subunit
RS: replicative senescence
SASP: senescence‐associated secretory phenotype
SICCs: senescence‐induced CTCF clusters
SIPS: stress‐induced premature senescence
STED: stimulated emission depletion microscopy
TAD: topologically associating domain
TIS: therapy‐induced senescence

## 1 INTRODUCTION

From the onset of development until late‐life stages, human cells encounter multiple signaling and stress cues. Many of these can lead to the induction of senescent phenotypes that commit cells to an irreversible growth arrest and are inextricably linked with ageing (Gorgoulis et al., 2019; López‐Otín et al., 2013; Schmeer et al., 2019). In fact, clearing senescent cells in vivo leads to prolonged health and lifespan (Baker et al., 2011, 2016; Wang et al., 2021, 2022). Depending on the initial trigger, senescent responses can be grouped into various types like replicative senescence (RS) occurring via telomere attrition, oncogene induced‐senescence (OIS) due to oncogenic activation, stress‐induced premature senescence (SIPS) due to oxidative stress, and DNA damage or therapy‐induced senescence (TIS) following chemo‐/radiotherapy (Campisi, 2013). All give rise to distinct gene expression programs, which however converge to an underlying transcriptional signature associated with cell cycle control and transcriptional remodeling (Hernandez‐Segura et al., 2017).

Apart from the pronounced cell cycle arrest, there are different genomic hallmarks of the commitment to senescence. For example, models of OIS show formation of large senescence‐associated heterochromatic foci (SAHFs) (Narita et al., 2003), which involve the dissociation of heterochromatin from the lamina, the redistribution of Lamin B1 (Sadaie et al., 2013; Shah et al., 2013) and nuclear pore components (Boumendil et al., 2019), as well as an interplay between DNMT1 and HMGA2 (Sati et al., 2020). These effects are also reflected on changes in the three‐dimensional (3D) organization of chromosomes (Chandra et al., 2015; Sati et al., 2020), with many have also being recorded in a model of DNA damage‐induced senescence (Zhang et al., 2021).

In RS, DNMT1 is linked to focal hypomethylation (Cruickshanks et al., 2013), and HMGB (rather than HMGA) proteins seem to play a central role as they are quantitatively depleted from senescent cell nuclei (Papantonis, 2021). The loss of HMGB1 was shown to affect both chromatin reorganization and mRNA splicing upon RS entry (Sofiadis et al., 2021), while that of HMGB2 was causal for heterochromatin imbalance and the formation of large senescence‐induced CTCF clusters (SICCs) (Zirkel et al., 2018). Cells maintained in RS long‐term (i.e., in “deep” senescence) display more pronounced changes in 3D genome organization, mostly compaction of chromosomal arms and changes between compartments of active and inactive chromatin (Criscione, Teo, et al., 2016) to suppress gene expression and activate transposable elements (De Cecco et al., 2013). This is in line with spurious (Sen et al., 2023) and accelerated transcription in senescence (Debès et al., 2023), with an overall compromised ability to transcribe (Zirkel et al., 2018), as well as with a transcription‐dependent reorganization of chromatin loops (Olan et al., 2020).

A key outcome of the senescent gene expression program is the production and secretion of a complex and cell type‐specific mixture of pro‐inflammatory factors: the senescence‐associated secretory phenotype (SASP) (Acosta et al., 2013; Kang et al., 2015; Laberge et al., 2015; Wiley et al., 2016). SASP factors act in an autocrine and a paracrine manner (Lopes‐Paciencia et al., 2019), and mediate both beneficial (e.g., wound healing) and detrimental effects of senescence (e.g., chronic inflammation and tumorigenesis) (Sun, Coppé, et al., 2018; Sun, Yu, et al., 2018). However, the production of SASP and other secondary signals (e.g., Notch in OIS; Teo et al., 2019) by senescent cells emerging in a population can both promote and limit senescence spread (Martin et al., 2023) in a manner that ultimately leads to a large heterogeneity of individual cell states (Chan et al., 2022; Teo et al., 2019; Wiley et al., 2017; Zirkel et al., 2018).

This pronounced heterogeneity, together with the asynchrony in senescence commitment by individual cells and the extended culture periods needed to reach replicative senescence, complicate studies of the core of the senescent program. Here, we address these caveats by the introduction of a novel and robust model of chemically induced senescence via the repurposing of the small molecule inhibitor ICM (Lee et al., 2014). We show that ICM induces senescence rapidly (within <6 days) and homogeneously in the popular fetal lung fibroblast (IMR90) cell model, while also constraining SASP production and its paracrine effects. We provide a comprehensive data resource by characterizing ICM‐induced senescence in order to facilitate its adoption by the broader community.

## 2 RESULTS

### 2.1 ICM induces a senescence‐like phenotype in human fibroblasts

**FIGURE 1:**
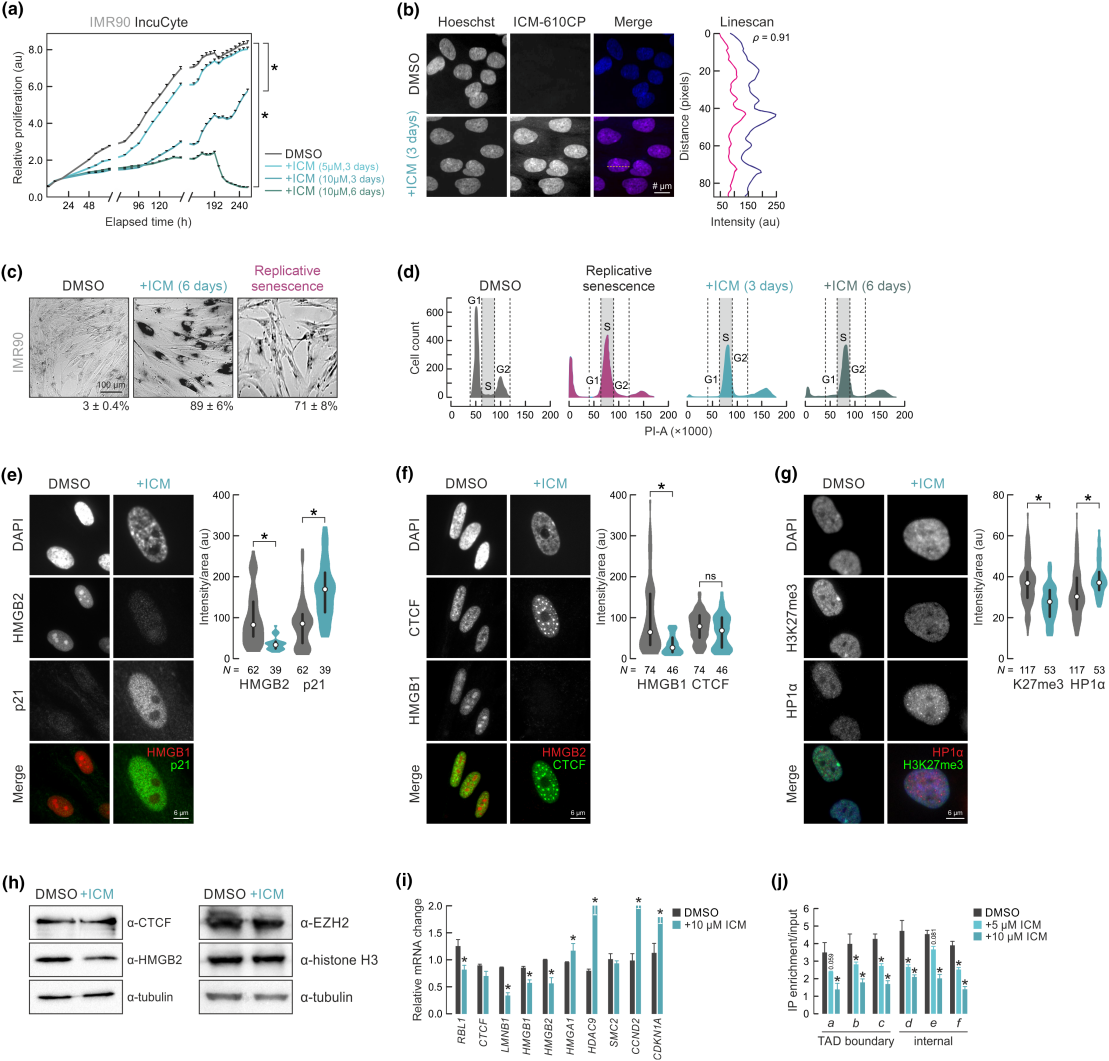
ICM treatment induces a senescent‐like phenotype. (a) Mean proliferation rates (±SEM from three independent replicates) of DMSO‐ and ICM‐treated IMR90 for 3–6 days using automated live‐cell imaging. *p < 0.01, unpaired two‐tailed Student's *t* test at 240 h. (b) Representative widefield images of proliferating (top) and 610CP‐C6‐ICM‐treated IMR90 (bottom) with DNA counterstained with Hoechst. The overlap between the two signals was assessed via a linescan. Bar, 5 μm. (c) Proliferating, ICM‐treated and senescent IMR90 assayed for β‐galactosidase activity. ICM‐treated and senescent cells appeared darker, indicative of their senescent state. (d) FACS cell cycle profiles of PI‐stained proliferating (DMSO), senescent or ICM‐treated IMR90 for 3 and 6 days. (e) Representative images of IMR90 showing treated or not with ICM for 6 days and immunostained for HMGB2 and p21. Bars, 6 μm. Violin plots (right) quantify changes in the levels of the two markers. *N*, number of cells analyzed per each condition. *p < 0.05, two‐tailed Wilcoxon‐Mann–Whitney test. (f) As in panel e, but immunostained for HMGB1 and CTCF. (g) As in panel e, but immunostained for HP1α and H3K27me3. (h) Western blot analysis of CTCF, HMGB2, EZH2 and histone H3 in proliferating (DMSO) and 6‐day ICM‐treated IMR90; α‐tubulin levels provide a loading control. (i) Mean mRNA levels (±SD from two independent isolates) of selected senescence marker genes in proliferating (DMSO) and 6‐day ICM‐treated IMR90. *p < 0.05, unpaired two‐tailed Student's *t* test. (j) Mean ChIP‐qPCR enrichment levels (±SD from two independent isolates) at selected genomic positions (a–f) in proliferating (DMSO) and 3‐ or 6‐day ICM‐treated IMR90. *p < 0.05, unpaired two‐tailed Student's *t* test.

Inflachromene (ICM) was initially discovered as a direct binder of HMGB1/‐B2 proteins in a broad screen of compounds, and characterized as a potent blocker of their cytoplasmic translocation and extracellular release. As a result, ICM restricted inflammatory phenotypes in vitro and in vivo to exert a neuroprotective effect (Lee et al., 2014). However, ICM was only tested in the neural context and for only up to 24 h. We subjected different isolates of fetal human lung fibroblasts (IMR90), one of the most popular models for senescence studies (Coppe et al., 2006; Demaria et al., 2014; Harley et al., 1990; Hayflick & Moorhead, 1961; Krtolica et al., 2001), to continuous exposure to different ICM concentrations. Treatment with 10 μM (but not 5 μM) ICM led to growth arrest within <4 days. Notably, removing ICM from the IMR90 growth medium after 6 days of treatment did not result in regrowth, while removal after 3 days of treatment did (Figure 1a). Such an effect of longer term ICM treatment was unexpected and prompted us to ask whether it actually induced a senescent‐like state to the cells.

First, we synthesized a 610CP‐C6‐tagged version of ICM (see Section 4) to verify that it indeed enters IMR90 nuclei and localizes to chromatin, where HMGBs reside; both could be confirmed microscopically (Figure 1b). Next, we stained control and 6‐day ICM‐treated cells for β‐galactosidase activity, a marker of senescence (Dimri et al., 1995). ICM‐treated cells stained essentially uniformly as SA‐β‐Gal‐positive, more than IMR90 from the same isolate driven to senescence by serial passaging, although they do not present with the same spindle‐like morphology (Figure 1c). FACS analysis showed that ICM treatment, already after 3 days, arrested IMR90 in late S‐phase (24.4% and 29.2% after 3‐ and 6‐day treatment, respectively, compared to 2.4% in DMSO‐treated cells), an effect comparable to the S‐phase accumulation seen in replicatively senescent cells (23.6%; Figure 1d).

It has been established that the nuclear depletion of HMGB1 and ‐B2 from the cell nucleus is a robust indicator of replicative senescence entry by different primary human cell types (Davalos et al., 2013; Sofiadis et al., 2021; Zirkel et al., 2018). We could show that pronounced nuclear loss of HMGBs is also achieved by ICM treatment of IMR90 (Figure 1e,f), together with the expected reduction in H3K27me3 levels (Figure 1g). These effects were coupled to the upregulation of senescence marker p21 (Figure 1e), the formation of senescence‐induced CTCF clusters (SICCs; Figure 1f), and the emergence of HP1α foci (Figure 1g), again, much like what has been recorded in RS (Zirkel et al., 2018). All these effects could be detected, albeit to a somewhat smaller extent, upon treatment with ICM for 3 days (Figure S1A,B), when growth arrest is not yet irreversibly committed to (Figure 1a). This suggests that HMGB1/‐B2 loss and SICC formation are early events on the path to senescence, as previously postulated (Zirkel et al., 2018). Moreover, ICM‐treated cells did not accumulate DNA damage as assessed by activated histone γH2A.X levels (Figure S1C,D).

Senescence induction was also reflected in changes at the protein and mRNA levels of all these factors, as well as of other known senescence‐regulated genes like *HDAC9*, *CCND2*, and *HMGA1* (Figure 1h,i). Finally, using ChIP‐qPCR we confirmed loss of HMGB2 chromatin binding upon ICM treatment from known cognate positions in RS‐IMR90 (Zirkel et al., 2018) at both topologically associating domain (TADs) boundaries and non‐boundary regions (Figure 1j). In summary, short‐term ICM treatment of IMR90 results in irreversible growth arrest, as well as in phenotypic changes likening those of RS.

### 2.2 Automated imaging and classification of nuclear features changes in ICM‐treated cells

**FIGURE 2:**
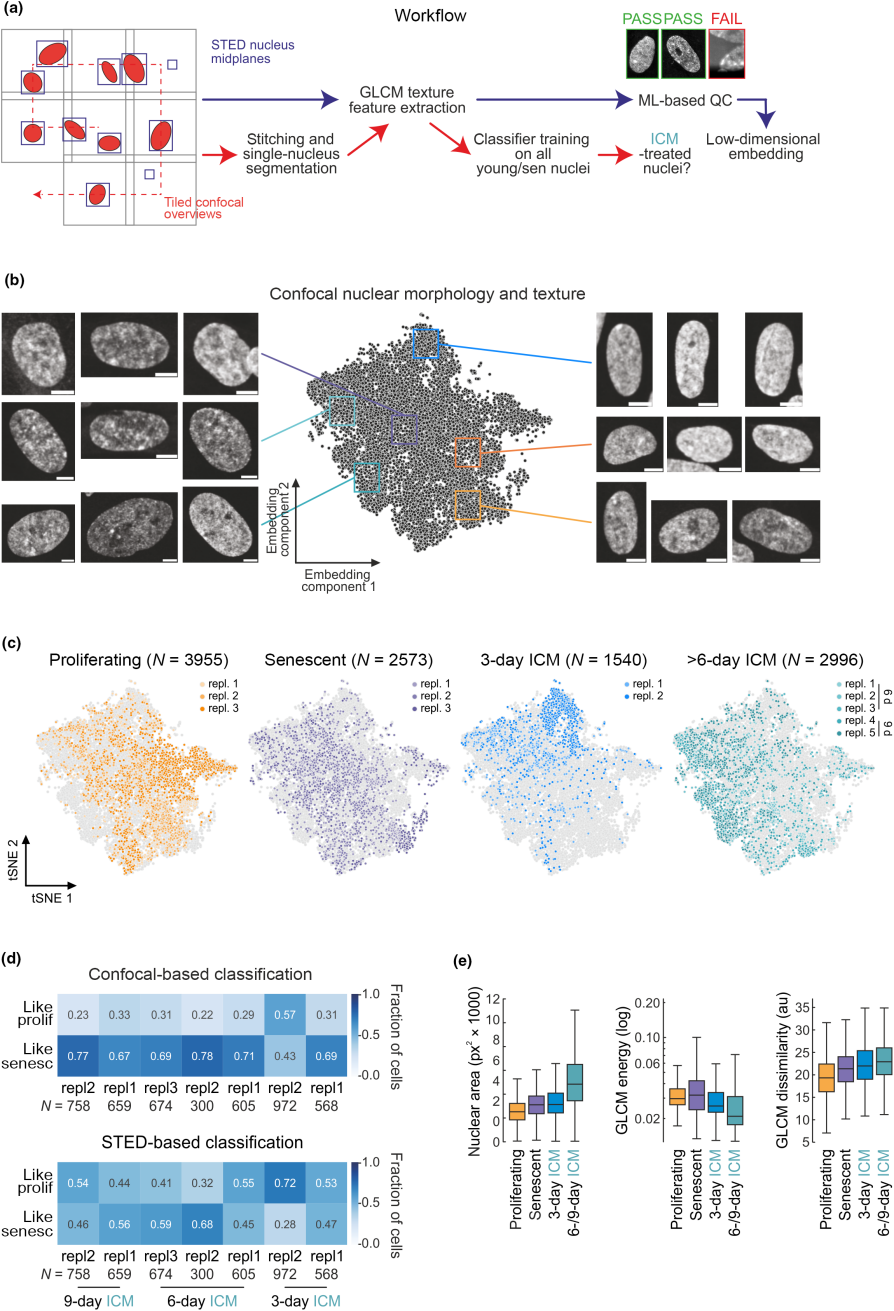
An automated classifier approach for assessing senescent cell nuclear morphology. (a) Overview of the automated imaging and analysis workflow. Coarse‐resolution confocal stacks were acquired in a tiled fashion and, once a nucleus of sufficient quality was detected, a mid‐plane STED image of it was also acquired. GLCM features were extracted from single nuclei from confocal (after tile stitching and maximum projection of z‐stacks) or STED images and used for downstream embedding and classification. (b) t‐SNE embedding of features extracted from confocal images with randomly selected example images from proliferating (orange), senescent (purple), and ICM‐treated cells (turquoise) shown. Bar, 5 μm. (c) As in panel b, but highlighting proliferating, senescent and 3‐ or 6‐day ICM‐treated cells of different replicates. (d) Confusion matrix showing the fraction of ICM‐treated cells classified as similar to proliferating or senescent cells using an SVM classifier trained on confocal‐ (top) or STED‐imaged nuclear features (bottom). (e) Box plots showing the distribution of nuclear size (left), GLCM energy values (in a 4‐pixel distance along the *x*‐axis; middle), and GLCM dissimilarity (in a 2‐pixel distance along the *y*‐axis; right) in confocal images of proliferating, senescent, 3‐ or 6‐/9‐day ICM‐treated cells.

Replicative senescence induces similar changes to the nuclear morphology of different primary human cell types, including size increase and a characteristic texture of DAPI chromatin staining (Sofiadis et al., 2021; Zirkel et al., 2018). Based on these observations, we reasoned that ICM‐treated cells could be classified as regards their senescence state via imaging of their nuclear features. To this end, we devised an automated imaging and classification workflow to process images of >11,000 IMR90 cells that were either proliferating (early‐passage), senescent or treated with ICM for 3–9 days. Our workflow used fixed cells counterstained by SiR‐DNA to visualize chromatin. Cells were identified and imaged via automated confocal imaging, while super‐resolution mid‐planes of individual nuclei were also captured using the platform's STED mode (Figure 2a). To discard erroneous detections, STED images of nuclei were filtered using a machine learning‐based quality control script achieving 95% precision in identifying “good” versus “bad quality” images (Figure 2a; see Section 4 for details).

We extracted GLCM texture (e.g., homogeneity, dissimilarity, energy, and angular second moment) and other features (e.g., area, eccentricity, and mean intensity) from each STED nuclear image that passed this QC and from full nuclei in confocal images, and used them for t‐SNE embedding (Figure 2b). In parallel, features from proliferating and replicatively senescent cells were used to train a Support Vector Machine (SVM) classifier and assess the extent to which cells treated with ICM for different numbers of days resembled RS ones. Using features extracted from confocal images, we recorded a broad range of nuclear phenotypes. t‐SNE embedding showed that most proliferating cells separate from senescent ones, albeit with considerable replicate mixing (Figure 2c). This was in line with previous single‐cell transcriptional profiling that identified senescent‐like cells in “young” populations and vice versa (Zirkel et al., 2018). We also saw 3‐day ICM‐treated nuclei predominantly clustering away from senescent and proliferating ones (likely representing an intermediate state), whereas 6‐ and 9‐day ICM‐treated IMR90 were proximal to senescent rather than to 3‐day or proliferating ones (Figure 2c). These patterns of separation also manifested in the SVM‐based classification of ICM‐treated cells. Both 6‐ and 9‐day‐treated IMR90 classified as senescent, whereas 3‐days ones scored as ambiguous (Figure 2d). No single extracted feature sufficed for exact classification, but increasing nuclear size, as well as changes in abstract features like GLCM energy and dissimilarity, appeared to better discriminate 6‐ and 9‐day ICM‐treated cells (Figure 2e). Notably, when STED images were used, sample‐to‐sample variation overshadowed phenotypic differences between proliferating and senescent nuclei resulting in the inconclusive classification of ICM‐treated cells (Figure 2d). We attribute this to the large effects that even small variance in, for example, DNA staining intensity can have on fine scale details captured by STED nanoscopy, as was also observed in a deep learning‐based study classifying senescence from images of nuclear‐stained cells, whereby coarse features provided more predictive power than finer scale ones (Heckenbach et al., 2022). Overall, we could deduce that ICM treatment of IMR90 produced nuclear features resembling those of senescent cells, most of which exhibit decreased heterogeneity (see replicate dispersal in Figure 2c).

### 2.3 ICM‐induced gene expression changes resemble replicative senescence

**FIGURE 3:**
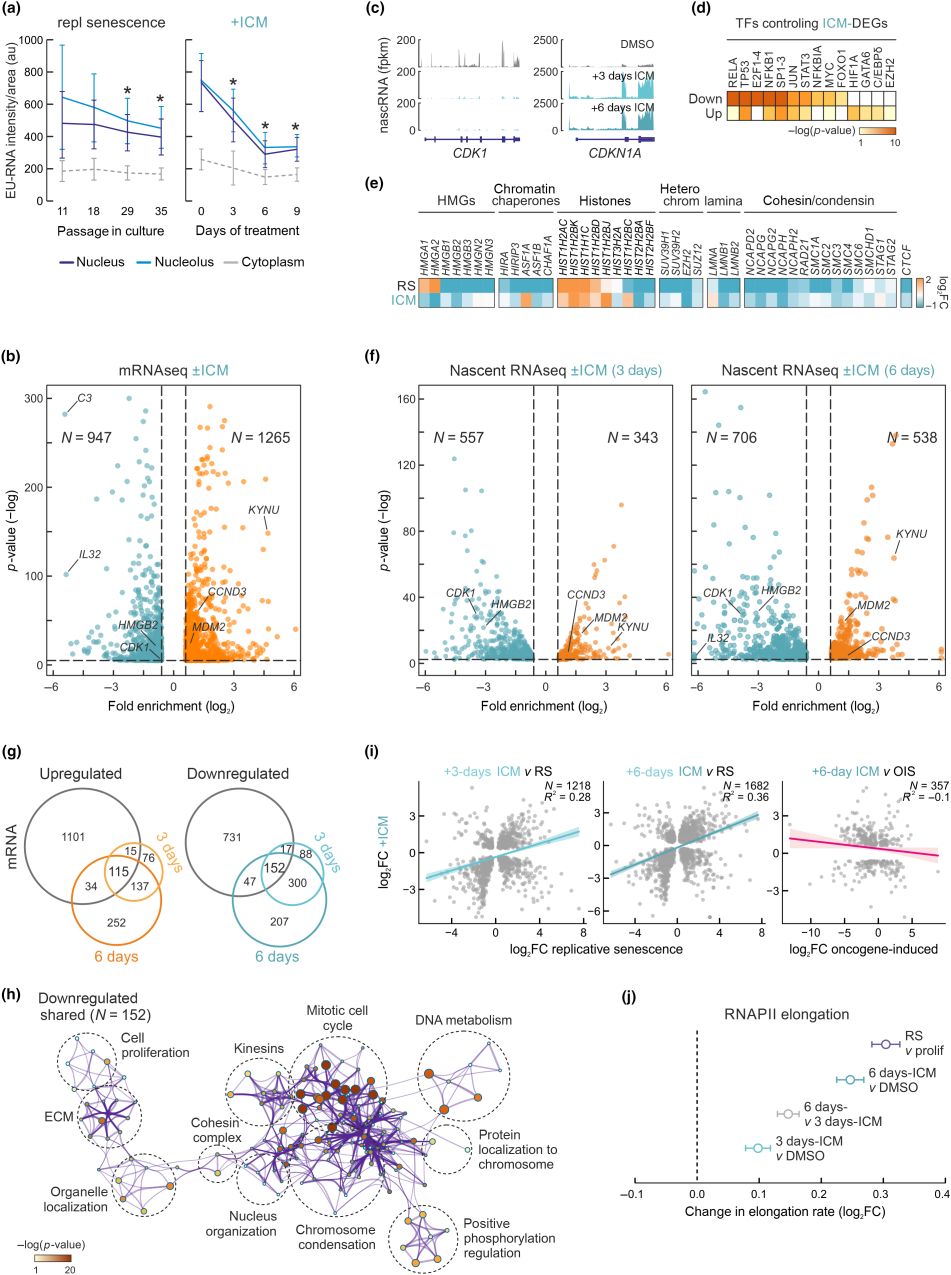
Transcriptional changes in ICM‐treated human lung fibroblasts. (a) Quantification of nascent EU‐RNA levels (by immunofluorescence) in IMR90 passaged into senescence (left) or treated with ICM (right). *Significantly different to starting nuclear/nucleolar levels; *p* < 0.05, unpaired two‐tailed Student's *t* test. (b) Volcano plot showing all differentially expressed mRNAs between proliferating and ICM‐treated IMR90. Significantly up‐ (orange; >0.6 log_2_FC) or down‐regulated ones (green; <−0.6 log_2_FC) are indicated. (c) RNA‐Seq profiles in the *CDK1* and *CDKN1A* locus from proliferating, 3‐ and 6‐day ICM‐treated IMR90s. (d) Heatmap showing transcription factors predicted to regulate genes from panel C based on motif enrichment. (e) Heatmap showing changes in mRNA levels upon senescence and ICM treatment of genes encoding selected chromatin‐associated factors. For each gene shown, statistically significant expression changes (log_2_FC) were recorded in at least one condition. (f) Volcano plot showing nascent RNA differences (fold enrichment) between 3 and 6 days of ICM treatment. Significantly up‐/downregulated genes are shown (>|0.6| log_2_‐fold change). *N* is the number of the genes in each group. (g) Venn diagrams of up‐/down‐regulated genes from ICM mRNA‐Seq and 3‐ or 6‐day ICM nascent RNA‐Seq. (h) GO term/pathway analysis of all commonly downregulated genes from panel g. (i) Comparison of differentially expressed genes between replicative senescence and 3 or 6 days of ICM treatment (left and middle panel, *R* ^2^ = 0.28 and 0.36, respectively) and oncogene induced senescence and ICM (right panel, *R* ^2^ = −0.1). *N* is the number of genes in each comparison. (j) Plot showing changes (log_2_FC ± SD) in mean RNAPII elongation rates calculated using nascent RNA‐Seq data.

We followed up the phenotypic characterization with gene expression profiling of ICM‐treated IMR90. First, based on previous data from RS, we would expect overall reduced RNA production if ICM‐cells had indeed committed to senescence. We measured this by incorporating EUTP into nascent RNA with a short pulse (7.5 min) and visualizing transcripts using an A488 fluorescent tag. Following quantification of signal intensity in the different cellular compartments, we saw an almost twofold drop in nuclear and nucleolar RNA levels by 6 days of ICM treatment, but only a modest decrease in labelled cytoplasmic RNA. This resembled the progressive drop seen in IMR90 passage into senescence (Figure 3a).

Despite this documented drop, senescence entry is characterized by a distinct program involving both up‐ and downregulated genes (Hernandez‐Segura et al., 2017; Sofiadis et al., 2021; Zirkel et al., 2018). We sequenced poly(A)^+^‐selected RNA (mRNA‐Seq) from two different IMR90 isolates treated with DMSO (control) or ICM for 6 days. We found ~950 and >1250 genes to be significantly (*p*_adj_ < 0.05, log_2_FC > |0.6|) differentially expressed (Figure 3b,c). These genes could be linked to gene ontology (GO) terms associated with landmark senescence pathways, such as mitotic cell cycle regulation and growth factor response (for downregulated ones; Figure S2A) or ECM organization and the p53 pathway (for upregulated ones; Figure S2B). Surprisingly, and contrary to the prominent pro‐inflammatory induction occurring in senescence (Coppé et al., 2010; Sofiadis et al., 2021), we found inflammatory activation via TNFα/NF‐κB and interleukins to be markedly suppressed by ICM treatment (Figure S2A), suggesting suppression of the SASP. This agreed with the anti‐inflammatory mode‐of‐action of ICM in neurons (Lee et al., 2014). Along these lines, a prediction of transcription factors (TFs) that regulate these differentially expressed genes via TTRUST (Han et al., 2018) revealed an expected enrichment for p53 and E2F‐family TFs for downregulated genes, but also strong enrichment of NF‐κB subunits (RELA, NFKB1) and co‐regulators (NFKBIA) involved in the inflammatory response (Figure 3d). Also, chromatin‐associated gene markers known to be regulated upon RS entry were similarly affected by ICM treatment with the notable exception of HMGA1 and A2, which have been implicated in the induction of heterochromatic foci in senescence and SASP regulation (Doubleday et al., 2020; Narita et al., 2006; Parry et al., 2018; Sati et al., 2020; Figure 3e).

Given that replicative senescence is a gene expression program predominantly regulated at the level of transcription (Sofiadis et al., 2021), we also sequenced and analyzed nascent RNA profiles from 3‐ and 6‐day ICM‐treated IMR90 via our “factory” RNA‐Seq approach (Caudron‐Herger et al., 2015). Using the same cutoffs as before, but analyzing intronic RNA levels (reflecting direct transcriptional changes), we identified 343 and 557 up‐ and downregulated genes, respectively, at 3 days post‐ICM treatment. These numbers increased to 538 and 706 after 6 days of treatment (Figure 3f). Despite this increase, the majority of 3‐day differentially expressed genes (i.e., 74% of up‐ and 81% of downregulated genes) were also identified at the 6‐day mark (Figure 3g) in line with the high convergence between the two time points (Figure S3A). Both sets associated with GO terms characteristic of RS induction (e.g., mitotic cell cycle, p53 pathway, and telomere organization; Figures S3B,C and S4A,B) and highly similar to those obtained by mRNA‐Seq analysis (Figure S2A,B). TFs predicted to control the 3‐ and 6‐day ICM‐regulated genes were also similar, with p53 and E2F‐family factors being most enriched (Figures S3D and S4C). However, there was a relative de‐enrichment for NF‐κB‐regulated downregulated genes at both time points, indicating that their suppression upon ICM treatment might not be exclusively transcriptional. Indeed, by looking at the overlap between differentially expressed mRNAs and nascent RNAs, only about 28% of up‐ or of downregulated nascent transcripts were also regulated at the messenger level, while >1000 up‐ and >700 downregulated mRNAs did not qualify as differentially expressed in factory RNA‐Seq data (Figure 3g). Some part of this can be attributed to a difference in approach and analysis (exon‐ vs. intron‐level quantification), but likely also points to posttranscriptional regulation (e.g., changes in mRNA stability). Still, the 152 commonly downregulated genes in the three datasets associated with the expected GO terms and pathways (Figure 3h).

Next, we used publicly‐available RNA‐Seq data from replicative (Rai et al., 2014) and oncogene‐induced senescence in IMR90 (Hernandez‐Segura et al., 2017), as well as a signature deduced from different types of senescence (Hernandez‐Segura et al., 2017) for a comparison to data from 3‐ and 6‐day ICM‐treated cells. We found a robust positive correlation with RS differentially expressed genes (*R* ^2^ = 0.28 and 0.36 for 3 and 6 days, respectively; Figure 3i) and with the consensus signature (*R* ^2^ = 0.84; Figure S5A), but no correlation with OIS (*R* ^2^ = −0.1; Figure 3i). This agrees with our phenotypic characterization showing resemblance of ICM‐induced senescence and RS, with a key discrepancy concerning inflammatory gene activation. We therefore compared RS with ICM mRNA‐Seq (wherein pro‐inflammatory gene suppression was most apparent; Figure S2A) to address this. The 175 genes strongly upregulated in RS, but suppressed by ICM treatment, were strongly associated with the pro‐inflammatory response and the SASP (Figure S5A,B).

Moreover, we wanted to address the extent to which ICM treatment produced effects that can be attributed to HMGB1/‐B2 depletion. We therefore analyzed *HMGB1* (Sofiadis et al., 2021) and new *HMGB2* siRNA‐mediated knockdown RNA‐Seq data (Figure S5C,D). We found ICM‐induced gene expression changes correlating strongly (*R* ^2^ = 0.46) with those recorded upon *HMGB2*‐KD (Figure S5C), and moderately anticorrelating (*R* ^2^ = −0.27) with those induced by *HMGB1*‐KD (Figure S5D). Gene set enrichment analysis (GSEA) of the positively and negatively correlated genes in each comparison confirmed previous analyses. Namely that *HMGB2*‐KD suppressed pro‐inflammatory responses and induced mitotic arrest (Figure S5E) as would be expected from ICM. On the other hand, *HMGB1*‐KD induced inflammatory cascades in contrast to ICM, with which it positively correlated as regards a pronounced cell cycle arrest (Figure S5F). Therefore, ICM treatment appears to promote replicative arrest via the same gene expression changes caused by *HMGB1*/‐*B2*‐KD, while also suppressing pro‐inflammatory gene induction caused mostly via the loss of HMGB1.

Finally, we examined two more features of cellular ageing, the increase in RNAPII velocity in senescent cells (Debès et al., 2023) and the changes in methylation levels at six senescence‐predictive CpGs (Franzen et al., 2017). The former showed the expected acceleration of RNAPII (Figure 3j), while the latter showed no predictive power during a 12‐day ICM treatment in contrast to how it performs for RS (Figure S5G).

### 2.4 Comparison of replicative and ICM‐induced senescence at the single cell level

One key motivation behind the pursuit of this system of ICM‐triggered senescence was the need for a more synchronous and homogeneous induction of senescence in a given cell population, as the entry into RS is largely stochastic and heterogeneous at the level of individual cells (Zirkel et al., 2018; for RS in HUVECs). This is not only due to idiosyncratic cell‐intrinsic properties (e.g., telomere attrition), but also a result of complex paracrine signaling via the SASP (Coppé et al., 2010). Thus, we reasoned that the apparently SASPless ICM phenotype would produce homogeneously‐senescent populations within 6 days of treatment.

**FIGURE 4:**
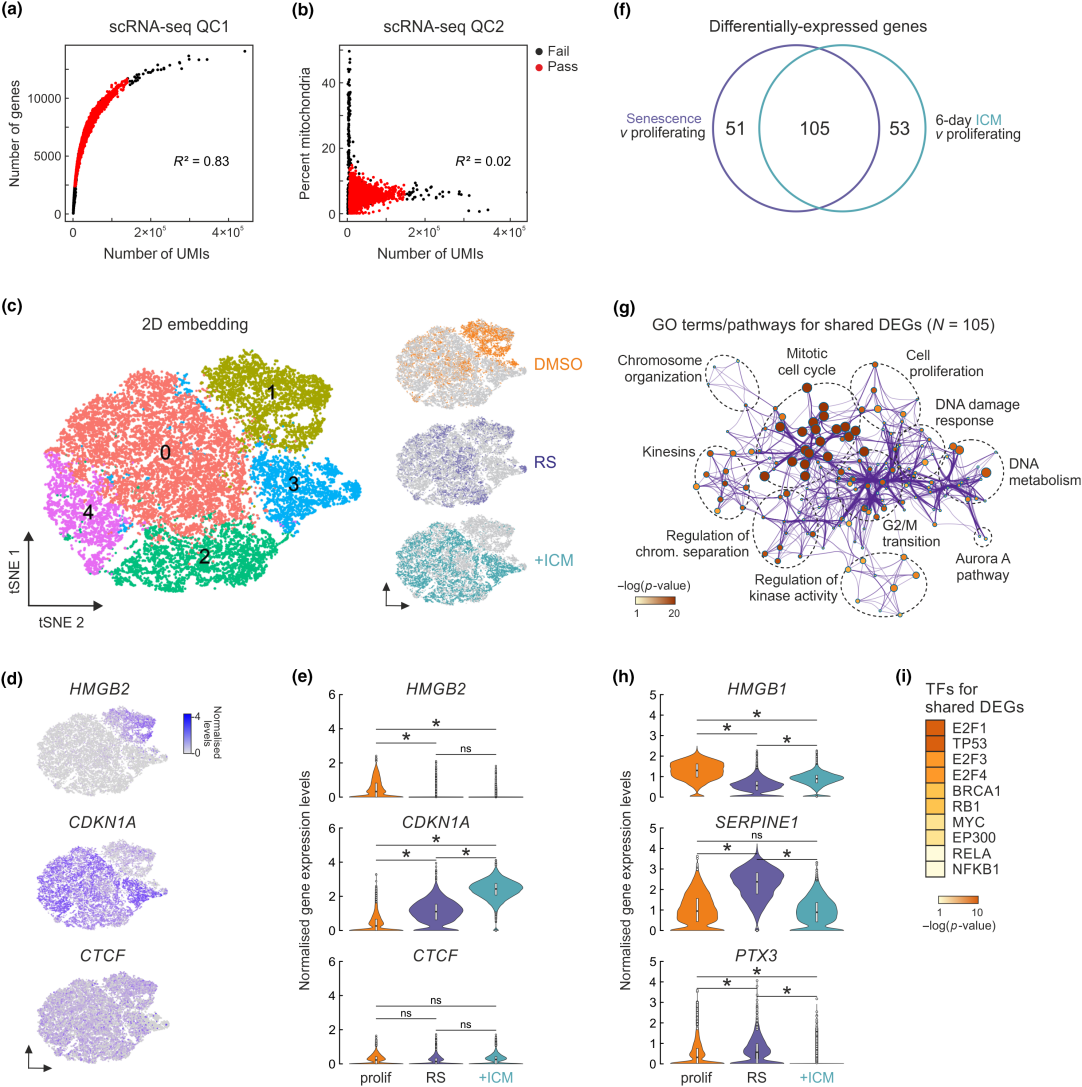
Single‐cell analysis of ICM‐induced transcriptomes. (a) Scatter plot of the number of unique molecular identifiers (UMIs) versus the number of detected genes in each cell analyzed. Cells that passed (red) or not (black) this quality filter and the calculated Spearman's correlation coefficient (*R* ^2^) are indicated. (b) As in panel a, but for the number of UMIs versus the percent of mitochondrial genes detected in each cell. (c) Left: t‐SNE embedding of gene expression profiles from 25,744 cells clustered in an unsupervised manner. Right: Projection of proliferating (DMSO), senescent (RS), and 6‐day ICM‐treated cells (+ICM) onto the t‐SNE map. (d) Projection of selected marker gene expression levels onto the t‐SNE map of panel c. (e) Violin plots showing expression level distribution of the marker genes from panel d in proliferating (prolif), senescent (RS), and 6‐day ICM‐treated cells (+ICM). *p < 0.01, Wilcoxon‐Mann–Whitney test. (f) Venn diagram showing the overlap of differentially expressed genes from senescent and ICM‐treated cells. (g) GO term/pathway analysis of the 105 shared differentially expressed genes from panel f. (h) As in panel e, but for exemplary SASP‐related genes. *p < 0.01, Wilcoxon–Mann–Whitney test. (i) Heatmap showing transcription factors predicted to regulate genes from panel f based on motif enrichment.

To test this, we generated single‐cell transcriptomic data from proliferating (DMSO‐treated), replicative senescent, and 6‐day ICM‐treated IMR90 from the same isolate. We interrogated a total of ~26,000 cells (8443 proliferating, 7947 senescent, and 9354 ICM‐treated) using 3′ end single‐cell RNA‐Seq. Following analysis of >1.8 billion reads (mean coverage was >70,000 reads/cell, median number of genes detected was 5697 genes/cell), single‐cell transcriptomes that met standard quality controls (Figure 4a,b) were used for in unsupervised clustering. This produced five clusters, as reflected in t‐SNE embedding, of which one (Cluster 1) was almost exclusively populated by proliferating and one (Cluster 3) exclusively by ICM‐treated cells (Figure 4c). In accordance to RS heterogeneity (Zirkel et al., 2018), some proliferating cells clustered among senescent cells (mostly in cluster 0) and some senescent cells mixed with proliferating ones (in cluster 1). However, ICM‐treated cells only mixed with senescent ones (in clusters 0, 2, and 4) and showed overall less dispersion in t‐SNE plots (Figure 4c). This was also reflected in the distribution of known senescence markers. For example, *HMGB2* and *DNMT1* were expressed in proliferating cells of Cluster 1 only and essentially not at all in senescent and ICM‐treated cells alike, while *CDKN1A* expression was confined to senescent and ICM‐treated cells, the latter showing significantly higher activation (Figure 4d,e). Largely uniform and low *CTCF* expression levels (as most transcription factors are lowly expressed) provided a control (Figure 4d,e).

Next, we identified condition‐specific differentially expressed genes. Using a threshold of log_2_FC > |0.25| and comparing all proliferating with either senescent or ICM‐treated cells, we detected >150 differentially expressed genes per condition. Of these, two‐third (i.e., 105 genes; more than expected by chance, *p* < 0.01) were shared between senescent and ICM‐treated cells highlighting their convergence (Figure 4f) and associated with GO terms central to the senescent phenotype, like cell cycle regulation, chromosome organization, or DNA metabolism (Figure 4g). Notably, almost all of the 105 genes were included in the 267 commonly differentially regulated genes seen by bulk RNA‐Seq measurements (Figure 3h).

Lastly, we asked how SASP regulation manifested in this data. Looking among differentially expressed genes for markers identified in the SASP atlas (http://www.saspatlas.com), like *SERPINE1* and PTX3, or genes indirectly controlling SASP production like *HMGA2* (Boumendil et al., 2019), we found that they were significantly upregulated across senescent cells, but reduced to below control levels in ICM‐treated IMR90 (Figure 4h). This was also reflected in the de‐enrichment for genes regulated by RELA and NFKB1 in the 105 differentially expressed genes shared by ICM‐treated and senescent cells (Figure 4i) and agreed with our bulk RNA‐Seq (Figure S5A,B) and TTRUST analysis (Figure 3d). Thus, our single‐cell data also confirmed the senescence‐like features of ICM‐induced cell growth arrest, as well as the more homogeneous nature of the response in IMR90 compared to senescence entry by continuous passaging.

### 2.5 ICM‐induced changes to the proteome are transcriptionally driven

**FIGURE 5:**
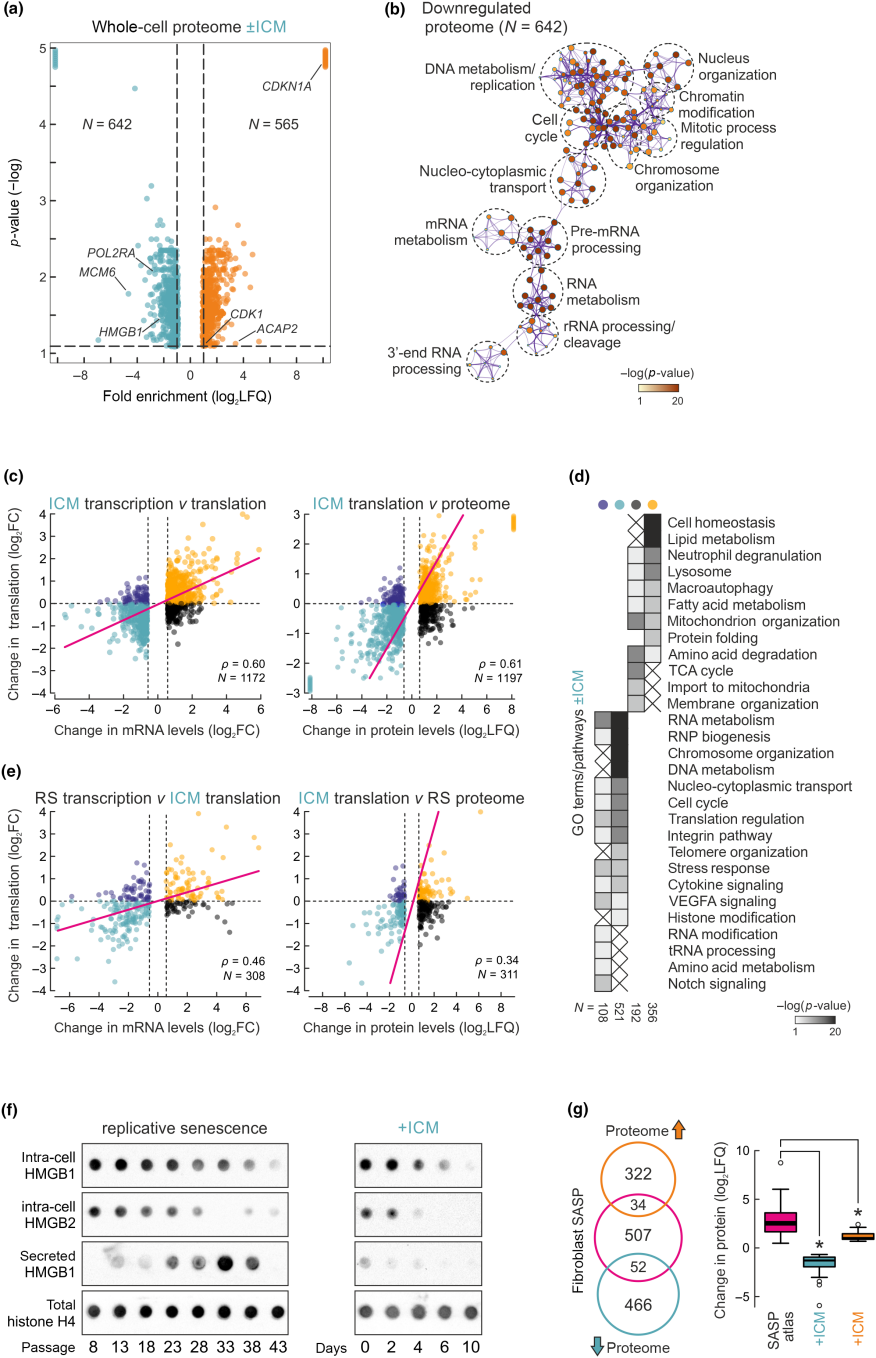
Proteomic changes induced by ICM treatment of IMR90. (a) Volcano plot showing whole proteome up‐ (orange, >0.6 log_2_LFQ) and downregulated proteins (turquoise, <−0.6 log_2_LFQ) upon 6 days of ICM treatment. The number of proteins (*N*) in each set is indicated. (b) GO term/pathways analysis of all downregulated proteins from panel a. (c) Left: Scatter plots showing correlation between mRNA‐Seq (transcription) and Ribo‐Seq levels (translation) of transcripts differentially expressed upon 6‐day ICM treatment. Right: Correlation between mRNA‐Seq and whole proteome levels for the same set of genes. The number of genes/proteins (*N*) in each set and Pearson's correlation coefficient (*ρ*) are indicated. (d) Heatmap showing GO terms/pathways associated with transcripts in the different quadrants of panel c (color‐coded the same way). The number of transcripts in each subgroup (*N*) is indicated. (e) As in panel c, but using differentially expressed genes from replicative senescence. (f) Dot blot showing intracellular HMGB1 and HMGB2 and secreted HMGB1 levels across passages (left panel) and days of ICM treatment. Histone H4 levels provide a control. (g) Left: Venn diagram showing up‐ and downregulated proteins from whole‐cell proteomics crossed with all known fibroblast SASP factors. Right: Box plot showing changes in SASP‐related protein levels (log_2_LFQ). *p < 0.01, unpaired two‐tailed Student's *t* test.

Despite strong indications from our gene expression analyses about the similarities between replicative and ICM‐induced senescence, it remained unclear whether the proteome also responded in the manner expected of senescent cells. To address this, we generated Ribo‐Seq and whole‐cell mass‐spec data from proliferating and ICM‐treated IMR90 in biological triplicates, and compared them to equivalent data generated previously for RS entry (Sofiadis et al., 2021). Whole‐cell proteome analysis after 6 days of ICM treatment revealed 565 significantly up‐ and 626 downregulated proteins (*p* < 0.05, log_2_LFQ > |0.6|; Figure 5a). GO term analysis of these differentially expressed proteins identified senescence hallmark pathways linked to both up‐ (e.g., stress response and p53 pathway) and downregulated ones (e.g., cell cycle, chromosome organization, and RNA metabolism) (Figure 5b and Figure S6A,B). This is in line with the differential analysis of RNA‐Seq data, with a TTRUST query predicting that the genes coding for downregulated proteins were controlled by p53, MYC, and E2F‐family TFs (Figure S6C), and with changes in the proteome of isolated IMR90 nuclei upon ICM treatment (Figure S7A–C).

We next focused on the regulation of translation upon ICM treatment. We previously used Ribo‐Seq to show that almost none of the changes in senescence‐related gene expression could be explained by changes in translation levels only (Sofiadis et al., 2021), and that no ribosome stalling, competition by upstream ORFs or translational deficiency could be detected (Papaspyropoulos et al., 2023). Thus, we generated Ribo‐Seq data for 6‐day ICM‐treated IMR90 and correlated them to matching mRNA‐Seq and whole‐cell proteome datasets. Much like what we observed for RS, all significant changes in translation were explained by an analogous change in transcript availability (Figure 5c). These transcripts were linked to key processes for senescence commitment like the downregulation of RNA metabolism, cell cycle regulation, and telomere organization (Figure 5d). There also were 300 transcripts “buffered” by translation (i.e., transcriptionally suppressed, but translationally boosted or vice versa), which could be implicated in pathways like RNA modification or Notch and VEGFA signaling (Figure 5c,d). These correlations remained largely unchanged when ICM mRNA‐Seq or Ribo‐Seq data were replaced by those generated in RS IMR90 (Figure 5e). Thus, the ICM‐induced expression program is predominantly regulated at the level of transcription, just like the one of RS (Sofiadis et al., 2021).

Interestingly, and in line with our mRNA‐Seq analysis, the expression of proteins involved in cytokine stimulation and the interferon response was suppressed by ICM treatment (Figure 5a and Figure S6A), and these transcripts were mostly found “buffered” when Ribo‐Seq was co‐considered (Figure 5d). Dot bot analysis of intracellular and extracellular levels of HMGB1 and B2 showed that, in contrast to what was observed during prolonged IMR90 passaging, HMGB1 is not released into the growth media as a pro‐inflammatory “alarmin” (Davalos et al., 2013) despite its apparent intracellular reduction (Figure 5f). ICM also constrained the TNFα‐induced secretome of IMR90 (Figure S6D). We assessed this more broadly by using a public catalogue of fibroblast SASP (http://www.saspatlas.com) and overlapping it with ICM‐induced proteome changes. Of ~600 bona fide SASP factors, <15% overlapped our data, with 52 being significantly down‐ and only 34 significantly upregulated (Figure 5g). However, even those upregulated by ICM, were not induced to the full extent observed in senescent fibroblasts (Figure 5g). Taken together, the above analyses confirm that ICM triggers a mostly SASP less senescence‐like phenotype.

### 2.6 ICM triggers 3D genome reorganization reminiscent of RS

**FIGURE 6:**
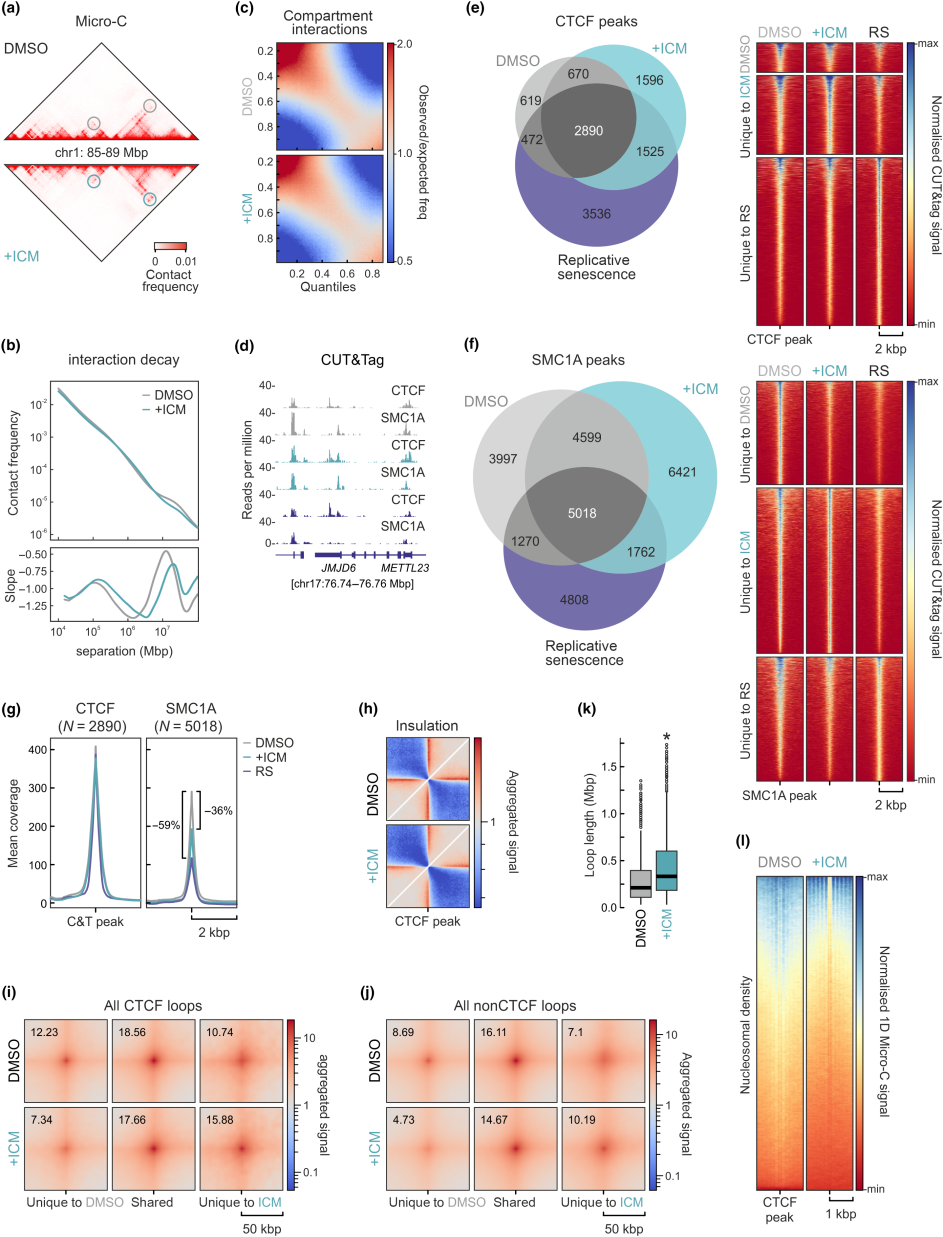
3D genome reorganization at 6 days post‐ICM treatment. (a) Heatmap showing Micro‐C contacts at 10‐kbp in an exemplary 4‐Mbp segments from chr1. Differences in loop formation between proliferating (DMSO) and ICM‐treated IMR90 (+ICM) are denoted (circles). (b) Plots showing decay of contact frequency as a function of genomic distance (top) and its first derivative (bottom) for proliferating and ICM‐treated cells. (c) Saddle plots showing contact distribution among and between inactive (top left corner) and active compartments (bottom right corner) in proliferating (DMSO) and ICM‐treated Micro‐C data (+ICM). (d) Representative genome browser views of CTCF and SMC1A CUT&Tag signal along a 100‐kbp region of chr17 from proliferating (grey), ICM‐treated (green) or senescent IMR90 (purple). (e) Left: Venn diagram showing the overlap of CTCF peaks (top 1%) in CUT&Tag data from proliferating, ICM‐treated, and senescent IMR90. Right: Heatmaps showing scaled CUT&Tag signal in the 4 kbp around the peaks. (f) As in panel e, but for SMC1A CUT&Tag data. (g) Line plots showing mean CTCF or SMC1A CUT&Tag signal coverage in the 4 kbp around all shared peaks from proliferating (grey), ICM‐treated (green) or senescent IMR90 (purple). (h) Insulation plot averaging Micro‐C contacts in the 600 kbp around CTCF peaks from proliferating (DMSO) and ICM‐treated IMR90 (+ICM). (i) Aggregate plots showing average Micro‐C signal in the 100 kbp around CTCF loop summits called at 5‐kbp resolution from unique to or shared by proliferating and ICM‐treated IMR90. (j) As in panel i, but for nonCTCF‐anchored loops. (k) Box plots showing the distribution of CTCF loop lengths in proliferating (DMSO) and ICM‐treated IMR90 (+ICM). *p < 0.01, Wilcoxon–Mann–Whitney test. (l) Heatmaps showing nucleosome distribution signal derived from Micro‐C data in the 2 kbp around CTCF motifs under CUT&Tag peaks from proliferating (DMSO) or ICM‐treated IMR90 (+ICM).

RS has been linked to extensive reorganization of 3D chromatin folding (Mizi et al., 2020), with entry into senescence already characterized by changes at the level of compartments and TADs (Sati et al., 2020; Zirkel et al., 2018). Therefore, as a last element in the characterization of ICM‐induced senescence, we addressed the extent of 3D genome reorganization after 6 days of treatment. We generated high‐resolution Micro‐C data (Hsieh et al., 2020) from proliferating (DMSO‐treated) and ICM‐treated IMR90. Replicates form each condition were sequenced to >1.1 billion read pairs generating maps with >614 and >711 million contact pairs for proliferating and ICM‐treated cells, respectively. Of these, >50% represented long‐range contacts (separated by >10 kbp; see Table S1 for details). As a result, we obtained dense 5‐kbp resolution contact maps with differences between conditions (Figure 6a).

In more detail, interaction decay plots showed reduced contact frequency at the Mbp scale, in parallel with increased frequency at sub‐Mbp separation distances (Figure 6b). The former is reminiscent of the “better compartment definition” we previously observed in lower resolution Hi‐C data from senescent IMR90 and HUVECs (Zirkel et al., 2018), and was corroborated by reduced inter‐compartmental interactions in ICM‐treated cells (Figure 6c). This can be explained in part by the ICM‐induced transcriptional suppression (Figure 3a) leading to chromatin compaction at the sub‐TAD level (Criscione, De Cecco, et al., 2016).

We also generated CUT&Tag data for the two key architectural factors giving rise to chromatin loops (Figure 6a), the insulator protein CTCF and the ring‐shaped cohesin complex via its SMC1A subunit (Hansen et al., 2017). Analysis of CTCF CUT&Tag returned 4652 CTCF peaks in proliferating, 8440 in senescent, and 6666 in ICM‐treated IMR90 (using the top 1% of peaks and filtering for the presence of a consensus CTCF motif under each peak; see Section 4) (Figure 6d,e). For SMC1A, 14,884 peaks were called in proliferating cells, 12,928 in senescent, and 17,810 in ICM‐treated ones (Figure 6d,f). Overall, ICM CTCF peaks overlapped more peaks from senescent rather than proliferating IMR90, but the converse was true for SMC1A (Figure 6e,f). Nevertheless, inspection of signal distribution in genome browser tracks and in heatmaps showed that there were indeed numerous strong CTCF peaks emerging in both ICM‐induced and replicative senescence, while for SMC1A this mostly held true in ICM‐treated cells (Figure 6d–f). However, SMC1A signal at shared CTCF peaks was reduced by at least 36% in ICM‐induced as well as in RS cells (Figure 6g).

The emergence of condition‐specific CTCF‐ and cohesin‐occupied positions along IMR90 chromosomes in combination with reduced cohesin levels led to a decrease in overall contact insulation around CTCF peaks (Figure 6h). This effect should translate into formation of condition‐specific loops from the 22,871 and 14,995 called in proliferating and ICM‐treated IMR90, respectively. After stratifying loops as CTCF‐ or nonCTCF‐anchored, we indeed found 613 CTCF (out of 1801; 34%) and 4303 nonCTCF loops (out of 13,194; 33%) that were unique to ICM‐treated cells and showed increased contact frequency (Figure 6i,j). Notably, and despite the fewer loops called in ICM data, loop length was significantly increased following ICM treatment (Figure 6k). This again agreed with previous observations from Hi‐C and CTCF HiChIP data in RS (Zirkel et al., 2018), and may be linked to the formation of senescence‐induced CTCF clusters (SICCs). Last, we exploited the fact that Micro‐C signal contains information about nucleosome positioning (Hsieh et al., 2020) to examine their density genome‐wide. We plotted 1‐D Micro‐C signal around CTCF peaks and found that, despite an overall signal decrease in ICM‐induced cells, nucleosomes were positioned in better defined arrays following ICM treatment (Figure 6l). Nucleosome signal decrease is characteristic of senescence (Debès et al., 2023), while the more defined positioning is reminiscent of that seen after RNAPII depletion from human cells (Zhang et al., 2023). Thus, 3D genome reorganization in ICM‐induced senescence also aligns well with RS entry.

## 3 DISCUSSION

Senescence is essentially hard‐wired in the homeostatic program of proliferating cells grown in vivo and in vitro (Hayflick & Moorhead, 1961). However, its emergence in cell populations is nonhomogeneous and heavily influenced by various waves of paracrine signaling (Kirschner et al., 2020; Martin et al., 2023). This poses several issues when studying cell commitment to senescence. For example, the core transcriptional program responsible for senescence entry might be obscured by differential gene induction and silencing due to SASP and secondary Notch signaling in a cell population. Similarly, studying the temporal order of molecular events leading up to senescence commitment can be obscured by the nonsynchronous manner by which it occurs in individual cells.

Here, we present a phenotypic and multi‐omics characterization of senescence induced in the popular IMR90 cellular model following treatment with the small molecule inhibitor ICM. We showed that, within 6 days of treatment with 10 μM ICM, a senescence‐like state is stably and irreversibly instated in the cell population in a synchronous and largely homogeneous manner. Most notably, senescence emerges in the absence of apparent paracrine signaling by the SASP that turns on pro‐inflammatory genes (Coppé et al., 2010). However, at first glance this seems to contrast the original characterization of the ICM mode‐of‐action (Lee et al., 2014). ICM was indeed developed and selected for its robust anti‐inflammatory capacity. This stemmed from its presumed ability to constrain the release of HMGB proteins, especially HMGB1, from the cell nucleus by interfering with their posttranslational modification (Lee et al., 2014). HMGB1 has a pronounced role as an “alarmin” released from cells to trigger inflammation in its niche in vitro and in vivo (Davalos et al., 2013; Papantonis, 2021; Salminen et al., 2012; Vénéreau et al., 2015). ICM treatment constrained neuroinflammation in cell cultures and animal models (Lee et al., 2014) and HMGB1‐related secretion and signaling from pancreatic cells (Chung et al., 2019) much like it constrains the SASP in IMR90 in our hands. However, the compound was never tested for treatment periods longer than 24 h, which would have allowed its full effects to deploy—namely, the synchronous and near‐complete depletion of both HMGB1 and B2 from nuclei, but without their apparent rerouting to the secretory pathway. Thereby, ICM does permanently restrict HMGBs in the nucleus, but can block secretion in favor of degradation, likely via the crosstalk of HMGB1 with the autophagy pathway (Kim et al., 2018). It would, thus, be interesting to eventually examine multi‐tissue effects following systematic in vivo ICM administration in animal models in more detail, while factoring in our observations from IMR90.

Having said this, ICM does produce some phenotypic and molecular effects that do not fully align with those of replicative senescence. Our single‐cell imaging and RNA‐Seq data support the resemblance of ICM‐treated IMR90 with those reaching senescence by passaging, but ICM treatment does not, for instance, give rise to a typical spindle‐like cell morphology of senescent cells, lead to the accumulation of DNA damage or to a senescence‐specific methylation signature. Such discrepancies discriminate ICM‐induced from replicative senescence, although this is the type of cell ageing phenotype that ICM‐treated cells resemble the most.

In summary, we wished to share ICM‐induced senescence with the broader community as a new tool for accelerating research into the cell‐intrinsic core of the senescent transcriptional program and the different components that mediate its irreversible nature (or allow for its sporadic bypass). Such rapid and synchronous pharmacological induction of senescence, devoid of paracrine influence, might even serve as a controllable in vitro system for testing antiaging (Browder et al., 2022; Roux et al., 2022) or senolytic approaches (Chaib et al., 2022; Robbins et al., 2020), and we provide a comprehensive characterization of it.

## 4 METHODS

### 4.1 Cell culture and senescent assays

Single IMR90 isolates (I90‐83, passage 5; Coriell Biorepository) were continuously passaged at 37°C under 5% CO_2_ in Minimal Essential Medium l‐Glutamine without HEPES (MEM 1×) (Gibco™ Life Technologies GmbH, 31095052) supplemented with 10 % FBS (Life Technologies, 10500064), 1× (1%) MEM Non‐essential Amino Acid Solution without l‐glutamine (Sigma‐Aldrich, M7145‐100ML) and 1% Penicillin/Streptomycin (Gibco™ Life Technologies, 15140122). The senescent state of the cells was addressed by senescence‐associated β‐galactosidase assay (Cell Signaling) according to the manufacturer's instructions. Cells were driven into senescence either by continuously passaging them to replicative exhaustion or by using ICM (concentration and period of treatment is depicted on individual experiments). Cell proliferation was monitored using the Sartorius IncuCyte S3 Live‐Cell Analysis System and acquiring a picture every 8 h for a total of 11 days. Finally, DNA methylation at six selected CpG islands was measured by isolating genomic DNA at the different cell states and performing targeted pyrosequencing (Cygenia GmbH) as previously detailed (Franzen et al., 2017).

### 4.2 Immunofluorescence and image analysis

Cells treated with ICM‐C6‐610CP were cultured on coverslips for 3 days and DNA was subsequently stained with 5‐SiR‐Hoechst (Bucevičius et al., 2020) and fixed via incubation with 4% paraformaldehyde (PFA) in Dulbecco's Phosphate‐Buffered Saline (DPBS). For every other staining, cells grown on coverslips were fixed via incubation with 4% PFA in DPBS at RT for 10 min and then permeabilized with 0.5% Triton‐X in PBS for 10 min. Blocking was performed with 1% Bovine Serum Albumin (BSA) in PBS at RT for 1 h. Cells were then incubated with the primary antibody (diluted in 0.5% BSA/PBS) at RT for 1 h at the indicated dilution: mouse monoclonal anti‐HMGB1 (1:1000; Abcam ab190377‐1F3); rabbit polyclonal anti‐HMGB2 (1:1000; Abcam ab67282); rabbit polyclonal anti‐CTCF (1:500; Active motif 61311); rabbit polyclonal anti‐H3K27me3 (1:1000; Diagenode C15410069); rabbit polyclonal anti‐p21 (1:500; Abcam EPR362—ab109520). The primary antibody was washed with PBS twice for 5 min per wash. Cells were incubated with the secondary antibody (diluted in 0.5% BSA/PBS) at RT, in the dark for 1 h at the indicated dilution: anti‐rabbit Alexa488 (1:1000, Abcam ab150077); anti‐mouse Cy3 (1:1000, Abcam ab97035). Cells were then washed with PBS twice for 5 min per wash. ProLongTM Gold antifade reagent with DAPI (#P36931) was added to the cells. For visualizing nascent transcripts, cells were pre‐incubated with 2.5 mM 5‐ethynyl uridine (EU) for 40 min at 37°C in their growth medium, fixed and processed with the Click‐iT EdU chemistry kit (Thermo Fisher). For image acquisition, a widefield Leica DMI8 with an HCX PL APO 63×/1.40 (Oil) objective was used. The acquired images were subsequently analyzed with the FIJI software (Schindelin et al., 2012). Measurements of nuclear immunofluorescence signal were generated using a mask drawn on DAPI staining to define nuclear bounds. Background subtractions were then implemented to precisely determine the mean intensity per area of each immune‐detected protein.

### 4.3 Automated cell imaging and feature classification

50–70 × 10^3^ IMR90 cells were seeded onto coverslips in 6‐well plates and left to grow at 37°C, with 5% CO_2_ in MEM (M4655), supplemented with 1% penicillin/streptomycin (Pen/Strep, P4333) and 1% nonessential amino acids (M7145) all from Sigma Aldrich, and 10% fetal bovine serum (FBS, F7524, LOT: BCBX5319) for 24 h before fixation and staining. For ICM‐treated cells, we exchanged the medium to one supplemented with 7.5 μM ICM after 24 h and left them to grow for 3, 6 or 9 days before fixation, exchanging the medium daily. The cells treated with ICM for 6 and 9 days were split once and twice, respectively. Cells were then prepared for immunofluorescence as described above, but also incubated in a SiR‐DNA (Lukinavičius et al., 2015) staining solution (2 μM in PBS‐T) for 90 min at room temperature. Afterward, cells were rinsed and washed twice for 5 min with PBST. Finally, coverslips with cells were mounted onto glass slides in MOWIOL. Thirty minutes after mounting, coverslips were sealed with clear nail varnish and dried for 20 min at room temperature.

We acquired confocal and STED images on an on a 3D STED microscope system from Abberior Instruments (Göttingen, Germany) using a 100× UPlanSApo 1.4 NA oil immersion objective (Olympus, Japan) and pulsed 640 nm excitation and 775 nm depletion lasers. To acquire a large number of super resolution images in an unbiased fashion, we automated the operation of the microscope using the *specpy* Python Interface to the Imspector microscope control software. In our automation pipeline, we first continuously imaged confocal overview stacks with 20% overlap in a spiral. Following the acquisition of each overview, we detected nuclei in the maximum intensity projection of the tile and adjacent tiles (stitched based on their stage coordinates) via unsupervised clustering of pixels based on their intensities and Gaussian and Sobel filter responses using k‐Means followed by binary erosion of radius = 3 to remove small background detections. For all connected foreground regions, we acquired a STED image of the middle z‐plane before continuing with the next overview tile. All acquired images as well as microscope metadata were saved in custom HDF5‐based files during the automated acquisitions. Using this pipeline, we could run the microscope unsupervised for prolonged periods of time and we typically imaged each sample for 12–24 h.

To extract features from STED images, we proceeded as follows: For each STED detail image, we first normalized the intensities to the 0.025 and 0.995 quantiles and then performed a simple segmentation by Li thresholding on a strongly blurred (Gaussian blur with *σ* = 16 px) version of the image followed by removing small objects <512 px^2^ and filling holes smaller than 512 px^2^. Within the foreground area we calculated grey level co‐occurrence matrices (GLCMs) at distances ∈ {2, 4, 7, 12, 16} and angles ∈ {0, *π*/2} and calculated all summary statistics available in scikit‐image's greycoprops function (“contrast,” “dissimilarity,” “homogeneity,” “energy,” “correlation” and “ASM”) from slightly blurred (*σ* = 0.5 px) versions of the normalized images. In addition, we calculated the mean foreground intensity in both the original and normalized image, the standard deviation of the foreground intensity, the number of pixels of the segmented area, the low and high quantiles of raw image intensities used in normalization, as well as the image width and height and number of rows and columns composed wholly of zeroes (an indication of images acquired outside the scanner's field of view or shutdown of the detector due to too high light exposure, which we aimed to remove in a subsequent quality control step). To distinguish good STED images from erroneous detections during the automated imaging we used machine learning‐based quality control. We manually sorted 493 images as good (i.e., containing a single complete and in‐focus nucleus) or bad and trained a Random Forest classifier on their features (normalized to zero mean and unit variance). Using fivefold cross‐validation, we determined the probability threshold for the good class necessary to achieve 95% precision (true‐positive rate) and applied the classifier with this threshold to all uncategorized images. For the subsequent steps, we only used the images classified as good.

We then used the features of each cell (except auxiliary ones like the number of blank rows/columns or image size) and performed a two‐dimensional embedding using t‐SNE. Furthermore, we used the features of all young and senescent samples to train a SVM classifier and applied it to all treated samples to see whether they would be preferentially classified as young or senescent. However, in both approaches, we arrived at inconclusive results and saw a strong dependence on individual replicates. We reasoned that at the fine scale captured by STED nanoscopy, small changes, for example, in SiR‐DNA staining intensity, have a stronger effect on nuclear texture than senescence state. The code for our image analysis pipeline was implemented in Python using the *numpy*/*scipy* (Harris et al., 2020), scikit‐image (Van Der Walt et al., 2014), and scikit‐learn (Pedregosa et al., 2011) libraries and is freely available at https://github.com/hoerlteam/chromatin‐texture‐senescence/. Due to sample‐to‐sample variation seems to overshadow biological effects on the nanoscale in STED images, we decided to also analyze the confocal overview images we initially acquired as auxiliary data during the automated imaging. We extracted individual overview tiles from the combined HDF5 files, saved them as TIFF stacks and used BigStitcher (Hörl et al., 2019) to stitch (refining the stage positions recorded by the microscope) and fused them into one volume per acquisition run. We then used Cellpose to detect individual nuclei in a z‐maximum projection and calculated GLCM texture features and summary statistics (with distances ∈ {2, 4, 8, 16} px and angles ∈ {0, *π*/2}) as well as shape and intensity features (area, eccentricity, and mean intensity) in the z‐projection for each detected nucleus individually. We normalized intensities for each image to the (0.025, 0.998) quantiles and applied a small amount of Gaussian blur (*σ* = 0.5 px) before extracting features. We then proceeded to perform t‐SNE embedding as well as SVM‐based classification of ICM‐treated cells based on young and old samples as described for the STED data above.

### 4.4 RNA isolation, sequencing, and analysis

Proliferating, senescent and ICM‐treated IMR90s were harvested in TRIzol LS (Life Technologies) and total RNA was isolated and DNase I‐treated using the DirectZol RNA miniprep kit (Zymo Research). Following selection on poly (dT) beads, barcoded cDNA libraries were generated using the TruSeq RNA library Kit (Illumina) and were paired‐end sequenced to >50 million read pairs on a HiSeq4000 platform (Illumina). Default settings of STAR aligner (Dobin et al., 2013) were used to map the raw reads to human reference genome (hg19) and quantification of unique counts was done via *featureCounts* (Liao et al., 2014). The RUVs function of RUVseq (Risso et al., 2014) was used to further normalize the counts, prior to differential gene expression estimation using DESeq2 (Love et al., 2014). Genes with an FDR < 0.01 and an absolute (log_2_) fold change of >0.6 were deemed as differentially expressed. GO term enrichment plots were generated using Metascape (http://metascape.org/gp/index.html) (Zhou et al., 2019). For RNA that was later used for qPCR the isolation procedure was the same as the one described above. cDNA was synthesized with SuperScript™ II Reverse Transcriptase (Invitrogen™ Life Technologies, 18064071) and random primers (Sigma‐Aldrich, 11034731001) according to the manufacturer's protocol. Full list of primers used for qPCR can be found in Table S2. Finally, for analysis of nascent RNA in IMR90 the “factory RNA‐Seq” approach was applied on 5mil ICM‐treated cells (Melnik et al., 2016), RNA was isolated and sequenced as above, and intronic read counts were obtained and differentially analyzed for the two conditions using the iRNAseq package (Madsen et al., 2015). Differentially expressed genes from all our RNA‐Seq experiments are listed in Table S3.

For RNAPII elongation rates calculated from “factory” RNA‐Seq data, annotation files were downloaded from Ensembl (https://www.ensembl.org/; version hg19). The following filtering steps were applied on the intronic ENSEMBL annotation files. First, we removed overlapping regions between introns and exons to avoid confounding signals due to variation in splicing or transcription initiation and termination. Overlapping introns were merged to remove duplicated regions from the analysis. In the next step, we used STAR to detect splice junctions and compared them with the intronic regions. Introns with at least five split reads bridging the intron (that is, mapping to the flanking exons) per condition were kept for subsequent analyses. When splice junctions were detected within introns, we further subdivided those introns accordingly. The slope of the intronic coverage was calculated in these introns across all samples as described (Debès et al., 2023). To avoid artefacts due to the different numbers of introns used per sample, we always contrasted the same sets of introns for each comparison of different conditions, only using negative slopes across all samples. The Wilcoxon signed‐rank test with continuity correction was used for statistical testing in R.

### 4.5 Chromatin immunoprecipitation and qPCR

Proliferating and ICM‐treated IMRO90s were cultured to 80% confluence in 15‐cm plates and they were cross‐linked in 15 mM EGS/PBS (ethylene glycol bis(succinimidyl succinate); Thermo) for 20 min at room temperature, followed by fixation for 40 min at 4°C in 1% PFA. Cells were then processed with the ChIP‐IT High Sensitivity kit (Active motif) according to the manufacturer's instructions. Chromatin was sheared to 200–500 bp fragments via sonication using a Bioruptor Plus (25 cycles, 30 s *on*/30 s *off*, high input), immunoprecipitation was done using 4 μg of anti‐HMGB2 antibody (Abcam ab67282) to approx. 30 μg of chromatin and the samples were incubated overnight in a rotor at 4°C. DNA was captured on protein A/G agarose beads and purified using the ChIP DNA Clean & Concentrator kit (Zymo) and used for qPCR. Oligos used in qPCR are listed in Table S4.

### 4.6 Ribo‐Seq and data analysis

High‐throughput ribosome profiling (Ribo‐Seq) on proliferating, senescent and ICM‐treated IMR90s was performed in collaboration with EIRNA Bio Ltd (https://eirnabio.com) according to their established protocol (Ivanov et al., 2018). Three independent replicas of proliferating, senescent or ICM‐treated IMR90 were grown, harvested in ice‐cold polysome isolation buffer supplemented with cycloheximide, and shipped to Ribomaps for further processing and library preparation. Roughly 15% of each lysate was kept for RNA isolation and used for RNA‐Seq of poly(A)‐enriched fractions on a HiSeq2500 platform (Illumina). After sequencing of both Ribo‐ and mRNA‐Seq libraries, the per base sequencing quality of each replicate passed the quality threshold, raw read counts were assigned to each protein‐coding open reading frame (CDS) for Ribo‐Seq and to each transcript for mRNA‐Seq, and replicate correlations were tested. Read length distribution for Ribo‐Seq datasets fell within the expected range (25–35 nt), showing strong periodic signals and an enrichment in annotated CDSs. For mRNA‐Seq, read lengths ranged between 47 and 51 nt and distributed uniformly across transcripts. For differential gene expression analysis, anota2seq (Oertlin et al., 2019) was used. Changes in Ribo‐Seq data depict changes in the ribosome occupancy of the annotated protein‐coding CDS, and thus, only ribosome‐protected fragments that map to the CDS were used in the analysis. VST normalized counts outputted using DESeq2 (Love et al., 2014) and inputted into anota2seq were used for all subsequent downstream analysis. Differences in genes that pass a default FDR threshold of 15% were considered regulated. Such significant differences are then categorized into one of the following three modes: (i) translational: Changes in Ribo‐Seq that are not explained by changes in RNA‐Seq and imply changes at the protein level are due to changes at the translational level; (ii) mRNA abundance: Matching changes in RNA‐Seq and Ribo‐Seq that infer changes at the protein level are predominantly induced by changes at the transcriptional level; (iii) *buffering*: changes in RNA‐Seq that are not explained by changes in Ribo‐Seq and suggest maintenance of constant protein levels induced by changes at the transcriptional level and vice versa. Differentially translated/buffered mRNAs from Ribo‐Seq experiments are listed in Table S5.

### 4.7 Protein extraction, western blotting, and mass spectrometry

Proliferating and ICM‐treated IMR90s (approx. 2 × 10^6^ per condition) were gently scraped off 15‐cm dishes. Cells were then pelleted for 5 min at 1200 rpm. The supernatant was discarded and pellets were lysed in 150 u of RIPA lysis buffer (20 mM Tris‐HCl pH 7.5, 150 mM NaCl, 1 mM EDTA pH 8.0, 1 mM EGTA pH 8.0, 1% NP‐40, 1% sodium deoxycholate) containing 1× protease inhibitor cocktail (Roche) for 30 min on ice. Sample were then sonicated in low input for three cycles (30 s on/30 s off) and centrifuged for 15 min at >15,000 × *g*. Then the supernatant was collected and the protein concentration was measured using the Pierce BCA Protein Assay Kit (Thermo Fisher Scientific). Rabbit polyclonal anti‐HMGB2 (1:1000; Abcam ab67282); rabbit polyclonal anti‐CTCF (1:500; Active motif 61311); rabbit polyclonal anti‐EZH2 (1:500; Active motif 39901); rabbit polyclonal anti‐H3 (1:500; Abcam ab1791); mouse monoclonal anti‐tubulin (1:1000; Abcam ab7291) were used for blotting. For whole‐cell proteomics, protein extracts in RIPA buffer were analyzed by the CECAD proteomic core facility in biological triplicates on a Q‐Exactive Plus Orbitrap platform (Thermo Scientific) coupled to an EASY nLc 1000 UPLC system with column lengths of up to 50 cm. All proteins discovered via whole‐cell mass spectrometry are listed in Table S6. For mass spectrometry of fractionated nuclear and cytosolic IMR90 extracts, the same procedure was followed with the addition of a standard nuclear isolation prep as described (Zirkel et al., 2018), and all proteins discovered are listed in Table S7.

### 4.8 Cleavage under targets and tagmentation (CUT&Tag)

0.5 million cells were lifted from plates using Accutase, fixed with 0.3% PFA/PBS for 2 min at RT and then quenched with 0.125 M ice cold glycine for 5 min at RT. Samples were then processed according to manufacturer's instructions (Active Motif). Samples were paired‐end sequenced to obtain more than 5 × 10^6^ reads, which were then processed exactly as described in https://yezhengstat.github.io/CUTTag_tutorial/. Briefly, paired‐end reads were trimmed for adapter removal and mapped to human (hg38) and *Escherichia coli* reference genomes (ASM584v2) using Bowtie 2 (Langmead & Salzberg, 2012). *E. coli* mapped reads were then quantified and used for calibrating human‐mapped reads. Peak calling was performed using a multi‐FDR‐tryout method (FDR <0.01 to <0.1). For CTCF and SMC1A, an FDR <0.01 was selected and only CTCF peaks with a canonical CTCF motif were considered (Grant et al., 2011). Motif search was conducted by utilizing Fimo 5.4.1 of the MEME suite (https://meme‐suite.org/meme/doc/fimo.html) against a random Markov background model which was created by running the *fasta‐get‐markov* command of the aforementioned suite, on random sequences that corresponded to the length and the chromosome of the query CTCF peaks, for each sample. Heatmaps were generated using deepTools (Ramírez et al., 2014), while shared and condition‐specific CTCF and SMC1A peaks were called using signal in the 100 bp around the summit of each peak (as calculated via SEACR).

### 4.9 Micro‐C and data analysis

Micro‐C was performed using the Micro‐C v1.0 kit in collaboration with Dovetail Genomics as per manufacturer's instructions. Micro‐C libraries (at least three per each biological replicate) that passed QC criteria were pooled and paired‐end sequenced on a NovaSeq6000 platform (Illumina) to >600 million read pairs per replicate. Micro‐C contact matrices were produced using Dovetail Genomics pipeline (https://micro‐c.readthedocs.io/en/latest/fastq_to_bam.html). In brief, read pairs were mapped to human reference genome hg38 using BWA, after which low mapping quality (<40) reads and PCR duplicates were filtered out using the *MarkDuplicates* function in Picard tools (v2.20.7), and read coverage tracks (BigWig) were generated and normalized with the RPCG parameter using the *bamCoverage* function of deepTools2 v3.5.1 (Ramírez et al., 2014). Compartment boundaries for each sample corresponded to the 1 bp of adjacent bins on which compartment changed from A to B or from B to A. The interaction decay plot was created by *cooltools* 0.5.1 (https://cooltools.readthedocs.io/en/latest/notebooks/contacts_vs_distance.html). The eigenvalues, needed for the saddle plots, were computed with the *cooltools call‐compartments* command at 10‐kbp resolution and the expected interactions were computed with cooltools compute‐expected command at the same resolution. The saddle plot was created with cooltools compute‐saddle using 100 bins as described: https://cooltools.readthedocs.io/en/latest/notebooks/compartments_and_saddles.html. Finally, we used *coolpuppy* 0.9.5 (https://coolpuppy.readthedocs.io/en/latest/) to generate all aggregate plots. For loop calling, we used a multi‐tool (HiCCUPS, SIP, and mustache) and a multi‐resolution (5‐ and 10‐kbp) approach as previously described (Hsieh et al., 2020; Krietenstein et al., 2020). Loop lists coming from each of the three different tools and across the two resolutions were merged using the *pgltools* intersect command with a distance tolerance of 1 bp. This procedure results in considering loops that were called in adjacent bins across different resolutions or tools as being shared, while unique loops are considered those that exhibit a distance corresponding to at least one bin size (5‐ or 10‐kbp) across the different loop‐calling approaches. In cases of shared loops across the two resolutions, the 5 kb resolution coordinates were kept for further analysis. In order to find condition‐specific loops we furtherly annotated them with ICM‐specific CTCF peaks. To detect ICM enriched CTCF peaks, we furtherly filtered peaks based on the control and ICM CUT&TAG signal enclosed in regions around the summits of ICM CTCF peaks. In more detail, we extracted the control and ICM depth‐normalized CUT&TAG signal of regions 100 bp around the summits of peaks by utilizing the *multiBigwigSummary* command of Deeptools. The CTCF peaks that we considered in the downstream analysis were those that exhibited less than the mean control CUT&TAG signal with higher or equal to 1‐fold difference compared to the corresponding ICM signal. 2628 ICM CTCF peaks fulfilled these criteria and were furtherly used to annotate both control and ICM loops. All intersections were performed using pgltools intersect1D without any distance tolerance for CTCF anchors. We considered loops as CTCF‐associated when at least one of the anchors overlapped a CTCF peak of the subset described above. The rest of the loops were annotated as non‐CTCF. We furtherly divided the loops into condition‐specific and shared loops. Condition‐specific loops had at least one unique anchor. This analysis was done, as described before, by utilizing the *pgltools* intersect command with 1 bp tolerance distance for both the shared and the unique loops. All code for Micro‐C analysis can be found at https://github.com/shuzhangcourage/Micro‐C‐CUT‐tag/tree/v1.0.0.

### 4.10 Single‐cell RNA‐Seq

Proliferating, ICM‐treated, and senescent IMR90s (8 × 10^5^ cells/condition) were grown to 80% confluency, harvested with trypsin and froze at −80°C. Single‐cell RNA‐Seq was performed using the 10× Genomics kit in collaboration with Active Motif. Libraries that passed QC criteria were paired‐end sequenced to at least 250 million reads per library. All downstream analysis was performed using Seurat (Satija et al., 2015).

### 4.11 610CP‐C6‐ICM synthesis

Synthesis was performed as described previously with the minor modifications (Isomura et al., 2001). 0.25 g (1.15 mmol) tert‐butyl (6‐hydroxyhexyl) carbamate (Molecule **2** in the scheme below) was dissolved in 3.2 mL dichloromethane under an argon atmosphere and cooled to 0°C in an ice bath. Next, 242 μL (1.73 mmol) of triethylamine and 98 μL (1.26 mmol) of mesyl chloride were added. The reaction mixture was then allowed to warm to room temperature and stirred for 2 h. After the reaction was complete, it was washed with distilled water, saturated NaCl solution, dried over Na_2_SO_4_, filtered, and the solvent removed on a rotary evaporator yielding yellow oil (359 mg). The residue was dissolved in 4.8 mL of dry acetonitrile under argon, 863 mg (5.76 mmol) of sodium iodide added at room temperature; the mixture stirred for 18 h, and the solvent was then removed on a rotary evaporator. The residue was dissolved in 20 mL ethyl acetate, washed with saturated NaCl solution, dried over Na_2_SO_4_, filtered and the solvent removed on a rotary evaporator to obtain 321 mg of crude product as a yellow oil. Next, flash chromatography was performed in *n*‐hexane/EtOAc (91:9) to obtain tert‐butyl (6‐iodohexyl) carbamate (mol. **3**) 294.8 mg (0.90 mmol, 78.3%) as a colorless oil.

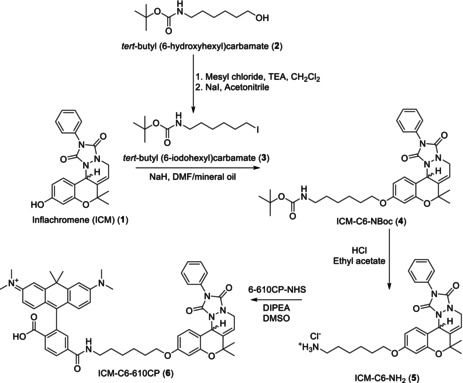

Next, 12.5 mg (33.1 μmol) inflachromene (Mol. **1**) was dissolved in 200 μL dry DMF under argon, cooled to 0°C, and 1.7 mg (42.5 μmol) of NaH (60% in mineral oil) added followed by stirring for 10 min. Next, 13 mg (40 μmol) tert‐butyl (6‐iodohexyl) carbamate (Mol. **3**) in 40 μL dry DMF was added dropwise, and the mixture stirred overnight at room temperature. After the reaction was complete, 30 μL of distilled water were added, the reaction mixture diluted with 2 mL saturated aq. NaCl solution and extracted with dichloromethane. The combined organic phase was washed with saturated NaCl solution, dried over Na_2_SO_4_, filtered, and the solvent removed on a rotary evaporator to obtain 26.7 mg of crude product as a yellow oil. Next, Flash chromatography was performed using Biotage HC Duo silica column and 15%–50% EtOAc in *n*‐Hexane gradient to obtain 7.5 mg (13.0 μmol, 39.3% yield) ICM‐C6‐NBoc (Mol. **4**) as a pale yellow solid.

2.0 mg (3.47 μmol) ICM‐C6‐NBoc (Mol. **4**) was dissolved in 17.3 μL of dry ethyl acetate under argon, cooled to 0°C using an ice bath, and 34.7 μL (34.68 μmol) of 1 M HCl in ethyl acetate were added followed by stirring for 6 h at room temperature. After the reaction was complete, the solvent was removed on the Speedvac to obtain 0.7 mg of yellow‐green oil and further HPLC purified using Interchim puriFlash® C18‐AQ 5 μm, 21.2 × 250 mm column with a solvent gradient from water with 0.1% TFA to acetonitrile, and obtained ICM‐C6‐NH2 (**5**) as a white solid (0.18 mg, 0.35 μmol, 10% yield).

Finally, 144 μg (255 nmol) 6‐610CP‐NHS was dissolved in 25 μL dry DMSO under argon. Next, 3.5 μL (2.04 μmol) 10% (v/v) DIPEA in dry DMSO and 180 μg (305 nmol) ICM‐C6‐NH2 (Mol. **5**) in 25 μL dry DMSO were added, and the reaction mixture stirred for 3 h at room temperature. After the reaction was complete, the mixture was frozen, lyophilized and separated using preparative HPLC: Interchim puriFlash® C18‐AQ 5 μm, 21.2 × 250 mm column with a solvent gradient from water with 0.1% TFA to acetonitrile, obtaining 160 μg (175 nmol, 69% yield) ICM‐C6‐610CP (Mol. **6**) as a blue solid. Compound purity characterization in shown in Figure S8.

### 4.12 Statistical testing

*p*‐values associated with Student's *t* tests, Fischer's exact tests and with the Wilcoxon–Mann–Whitney tests were calculated using GraphPad (https://graphpad.com/). Unless otherwise stated, *p‐*values <0.01 were deemed as statistically significant.

## AUTHOR CONTRIBUTIONS

SP, KS, NJ, IP, and AZ performed experiments; AS, VV‐M, YZ, NJ, and CN performed computational analyses; AD‐M analyzed single‐cell genomics data; AOC, GLO, JK, and AM generated and analyzed Ribo‐Seq data; DH, SN, HH, and HL generated imaging data and devised the classification pipeline; GLU produced the 610CP‐C6‐ICM compound; AnP and AB provided the RNAPII speed calculations; WW provided methylation data; AP conceived the study; SP and AP wrote the manuscript with input from all co‐authors.

## FUNDING INFORMATION

This work was supported by the German Research Foundation (DFG) via TRR81 (Project 109546710 awarded to AP), the SPP2202 Priority program (Projects 422389065 to AP, and 422857584 to HH and HL), the KFO5002 Clinical Research Unit (Project 426671079 to AP), as well as by the SFB1565 (Project 469281184 to AP). SP, KS, NJ, AD‐M, and VV‐M were also supported by the International Max Planck Research School for Genome Science, YZ by the International Max Planck Research School for Molecular Biology, and AS by Erasmus+ funds.

## CONFLICT OF INTEREST STATEMENT

WW is cofounder of Cygenia GmbH (www.cygenia.com) providing epigenetic senescence analysis services. Apart from this, the authors declare that they have no conflict of interest.

## Supporting information


Appendix S1

## Data Availability

All NGS data generated have been deposited to the NCBI Gene Expression Omnibus (GEO) under accession number GSE238256 (https://www.ncbi.nlm.nih.gov/geo/query/acc.cgi?acc=GSE238256), while all imaging data can be found on the OSF (https://osf.io/n7qxc/?view_only=067396ac892542fc98767205f2063613).

## References

[acel14083-bib-0001] Acosta, J. C. , Banito, A. , Wuestefeld, T. , Georgilis, A. , Janich, P. , Morton, J. P. , Athineos, D. , Kang, T. W. , Lasitschka, F. , Andrulis, M. , Pascual, G. , Morris, K. J. , Khan, S. , Jin, H. , Dharmalingam, G. , Snijders, A. P. , Carroll, T. , Capper, D. , Pritchard, C. , … Gil, J. (2013). A complex secretory program orchestrated by the inflammasome controls paracrine senescence. Nature Cell Biology, 15(8), 978–990. 23770676 10.1038/ncb2784 PMC3732483

[acel14083-bib-0002] Baker, D. J. , Childs, B. G. , Durik, M. , Wijers, M. E. , Sieben, C. J. , Zhong, J. , Saltness, R. A. , Jeganathan, K. B. , Verzosa, G. C. , Pezeshki, A. , Khazaie, K. , Miller, J. D. , & Van Deursen, J. M. (2016). Naturally occurring p16Ink4a‐positive cells shorten healthy lifespan. Nature, 530, 184–189. 26840489 10.1038/nature16932 PMC4845101

[acel14083-bib-0003] Baker, D. J. , Wijshake, T. , Tchkonia, T. , Lebrasseur, N. K. , Childs, B. G. , Van De Sluis, B. , Kirkland, J. L. , & Van Deursen, J. M. (2011). Clearance of p16Ink4a‐positive senescent cells delays ageing‐associated disorders. Nature, 479(7372), 232–236. 22048312 10.1038/nature10600 PMC3468323

[acel14083-bib-0004] Boumendil, C. , Hari, P. , Olsen, K. C. F. , Acosta, J. C. , & Bickmore, W. A. (2019). Nuclear pore density controls heterochromatin reorganization during senescence. Genes & Development, 33, 144–149. 10.1101/gad.321117.118 30692205 PMC6362808

[acel14083-bib-0005] Browder, K. C. , Reddy, P. , Yamamoto, M. , Haghani, A. , Guillen, I. G. , Sahu, S. , Wang, C. , Luque, Y. , Prieto, J. , Shi, L. , Shojima, K. , Hishida, T. , Lai, Z. , Li, Q. , Choudhury, F. K. , Wong, W. R. , Liang, Y. , Sangaraju, D. , Sandoval, W. , … Izpisua Belmonte, J. C. (2022). In vivo partial reprogramming alters age‐associated molecular changes during physiological aging in mice. Nature Aging, 2, 243–253. 37118377 10.1038/s43587-022-00183-2

[acel14083-bib-0006] Bucevičius, J. , Kostiuk, G. , Gerasimaitė, R. , Gilat, T. , & Lukinavičius, G. (2020). Enhancing the biocompatibility of rhodamine fluorescent probes by a neighbouring group effect. Chemical Science, 11, 7313–7323. 33777348 10.1039/d0sc02154g PMC7983176

[acel14083-bib-0007] Campisi, J. (2013). Aging, cellular senescence, and cancer. Annual Review of Physiology, 75, 685–705. 10.1146/annurev-physiol-030212-183653 PMC4166529 23140366

[acel14083-bib-0008] Caudron‐Herger, M. , Cook, P. R. , Rippe, K. , & Papantonis, A. (2015). Dissecting the nascent human transcriptome by analysing the RNA content of transcription factories. Nucleic Acids Research, 43, e95. 25897132 10.1093/nar/gkv390 PMC4538806

[acel14083-bib-0009] Chaib, S. , Tchkonia, T. , & Kirkland, J. L. (2022). Cellular senescence and senolytics: The path to the clinic. Nature Medicine, 28(8), 1556–1568. 10.1038/s41591-022-01923-y PMC9599677 35953721

[acel14083-bib-0010] Chan, M. , Yuan, H. , Soifer, I. , Maile, T. M. , Wang, R. Y. , Ireland, A. , O'Brien, J. , Goudeau, J. , Chan, L. , Vijay, T. , Freund, A. , Kenyon, C. , Bennett, B. , McAllister, F. , Kelley, D. R. , Roy, M. , Cohen, R. L. , Levinson, A. D. , Botstein, D. , & Hendrickson, D. G. (2022). Novel insights from a multiomics dissection of the Hayflick limit. eLife, 11, e70283. 10.7554/eLife.70283 35119359 PMC8933007

[acel14083-bib-0011] Chandra, T. , Ewels, P. A. , Schoenfelder, S. , Furlan‐Magaril, M. , Wingett, S. W. , Kirschner, K. , Thuret, J.‐Y. , Andrews, S. , Fraser, P. , & Reik, W. (2015). Global reorganization of the nuclear landscape in senescent cells. Cell Reports, 10, 471–483. 25640177 10.1016/j.celrep.2014.12.055 PMC4542308

[acel14083-bib-0012] Chung, H. , Hong, S. J. , Choi, S. W. , Koo, J. Y. , Kim, M. , Kim, H. J. , Park, S. B. , & Park, C. G. (2019). High mobility group box 1 secretion blockade results in the reduction of early pancreatic islet graft loss. Biochemical and Biophysical Research Communications, 514, 1081–1086. 10.1016/j.bbrc.2019.05.003 31097219

[acel14083-bib-0013] Coppé, J. P. , Desprez, P. Y. , Krtolica, A. , & Campisi, J. (2010). The senescence‐associated secretory phenotype: The dark side of tumor suppression. Annual Review of Pathology, 5, 99–118. 10.1146/annurev-pathol-121808-102144 PMC4166495 20078217

[acel14083-bib-0014] Coppe, J. P. , Kauser, K. , Campisi, J. , & Beauséjour, C. M. (2006). Secretion of vascular endothelial growth factor by primary human fibroblasts at senescence. The Journal of Biological Chemistry, 281, 29568–29574. 10.1074/jbc.M603307200 16880208

[acel14083-bib-0015] Criscione, S. W. , De Cecco, M. , Siranosian, B. , Zhang, Y. , Kreiling, J. A. , Sedivy, J. M. , & Neretti, N. (2016). Reorganization of chromosome architecture in replicative cellular senescence. Science Advances, 2, e1500882. 10.1126/sciadv.1500882 26989773 PMC4788486

[acel14083-bib-0016] Criscione, S. W. , Teo, Y. V. , & Neretti, N. (2016). The chromatin landscape of cellular senescence. Trends in Genetics, 32, 751–761. 27692431 10.1016/j.tig.2016.09.005 PMC5235059

[acel14083-bib-0017] Cruickshanks, H. A. , McBryan, T. , Nelson, D. M. , Vanderkraats, N. D. , Shah, P. P. , Van Tuyn, J. , Singh Rai, T. , Brock, C. , Donahue, G. , Dunican, D. S. , Drotar, M. E. , Meehan, R. R. , Edwards, J. R. , Berger, S. L. , & Adams, P. D. (2013). Senescent cells harbour features of the cancer epigenome. Nature Cell Biology, 15, 1495–1506. 24270890 10.1038/ncb2879 PMC4106249

[acel14083-bib-0018] Davalos, A. R. , Kawahara, M. , Malhotra, G. K. , Schaum, N. , Huang, J. , Ved, U. , Beausejour, C. M. , Coppe, J. P. , Rodier, F. , & Campisi, J. (2013). p53‐dependent release of alarmin HMGB1 is a central mediator of senescent phenotypes. The Journal of Cell Biology, 201, 613–629. 10.1083/jcb.201206006 23649808 PMC3653366

[acel14083-bib-0019] De Cecco, M. , Criscione, S. W. , Peckham, E. J. , Hillenmeyer, S. , Hamm, E. A. , Manivannan, J. , Peterson, A. L. , Kreiling, J. A. , Neretti, N. , & Sedivy, J. M. (2013). Genomes of replicatively senescent cells undergo global epigenetic changes leading to gene silencing and activation of transposable elements. Aging Cell, 12, 247–256. 10.1111/acel.12047 23360310 PMC3618682

[acel14083-bib-0020] Debès, C. , Papadakis, A. , Grönke, S. , Karalay, Ö. , Tain, L. S. , Mizi, A. , Nakamura, S. , Hahn, O. , Weigelt, C. , Josipovic, N. , Zirkel, A. , Brusius, I. , Sofiadis, K. , Lamprousi, M. , Lu, Y.‐X. , Huang, W. , Esmaillie, R. , Kubacki, T. , Späth, M. R. , … Beyer, A. (2023). Ageing‐associated changes in transcriptional elongation influence longevity. Nature, 616, 814–821. 37046086 10.1038/s41586-023-05922-y PMC10132977

[acel14083-bib-0021] Demaria, M. , Ohtani, N. , Youssef, S. A. , Rodier, F. , Toussaint, W. , Mitchell, J. R. , Laberge, R. M. , Vijg, J. , VanSteeg, H. , Dollé, M. E. T. , Hoeijmakers, J. H. J. , de Bruin, A. , Hara, E. , & Campisi, J. (2014). An essential role for senescent cells in optimal wound healing through secretion of PDGF‐AA. Developmental Cell, 31, 722–733. 10.1016/j.devcel.2014.11.012 25499914 PMC4349629

[acel14083-bib-0022] Dimri, G. P. , Lee, X. , Basile, G. , Acosta, M. , Scott, G. , Roskelley, C. , Medrano, E. E. , Linskens, M. , Rubelj, I. , Pereira‐Smith, O. , Peacocke, M. , & Campisi, J. (1995). A biomarker that identifies senescent human cells in culture and in aging skin in vivo. Proceedings of the National Academy of Sciences of the United States of America, 92, 9363–9367. 7568133 10.1073/pnas.92.20.9363 PMC40985

[acel14083-bib-0023] Dobin, A. , Davis, C. A. , Schlesinger, F. , Drenkow, J. , Zaleski, C. , Jha, S. , Batut, P. , Chaisson, M. , & Gingeras, T. R. (2013). STAR: Ultrafast universal RNA‐seq aligner. Bioinformatics, 29, 15–21. 23104886 10.1093/bioinformatics/bts635 PMC3530905

[acel14083-bib-0024] Doubleday, P. F. , Fornelli, L. , & Kelleher, N. L. (2020). Elucidating proteoform dynamics underlying the senescence associated secretory phenotype. Journal of Proteome Research, 19, 938–948. 10.1021/acs.jproteome.9b00739 31940439 PMC7032038

[acel14083-bib-0025] Franzen, J. , Zirkel, A. , Blake, J. , Rath, B. , Benes, V. , Papantonis, A. , & Wagner, W. (2017). Senescence‐associated DNA methylation is stochastically acquired in subpopulations of mesenchymal stem cells. Aging Cell, 16, 183–191. 10.1111/acel.12544 27785870 PMC5242294

[acel14083-bib-0026] Gorgoulis, V. , Adams, P. D. , Alimonti, A. , Bennett, D. C. , Bischof, O. , Bishop, C. , Campisi, J. , Collado, M. , Evangelou, K. , Ferbeyre, G. , Gil, J. , Hara, E. , Krizhanovsky, V. , Jurk, D. , Maier, A. B. , Narita, M. , Niedernhofer, L. , Passos, J. F. , Robbins, P. D. , … Demaria, M. (2019). Cellular senescence: Defining a path forward. Cell, 179, 813–827. 31675495 10.1016/j.cell.2019.10.005

[acel14083-bib-0027] Grant, C. E. , Bailey, T. L. , & Noble, W. S. (2011). FIMO: Scanning for occurrences of a given motif. Bioinformatics, 27, 1017–1018. 21330290 10.1093/bioinformatics/btr064 PMC3065696

[acel14083-bib-0028] Han, H. , Cho, J. W. , Lee, S. , Yun, A. , Kim, H. , Bae, D. , Yang, S. , Kim, C. Y. , Lee, M. , Kim, E. , Lee, S. , Kang, B. , Jeong, D. , Kim, Y. , Jeon, H. N. , Jung, H. , Nam, S. , Chung, M. , Kim, J. H. , & Lee, I. (2018). TRRUST v2: An expanded reference database of human and mouse transcriptional regulatory interactions. Nucleic Acids Research, 46, D380–D386. 10.1093/nar/gkx1013 29087512 PMC5753191

[acel14083-bib-0029] Hansen, A. S. , Pustova, I. , Cattoglio, C. , Tjian, R. , & Darzacq, X. (2017). CTCF and cohesin regulate chromatin loop stability with distinct dynamics. eLife, 6, e25776. 10.7554/eLife.25776 28467304 PMC5446243

[acel14083-bib-0030] Harley, C. B. , Futcher, A. B. , & Greider, C. W. (1990). Telomeres shorten during ageing of human fibroblasts. Nature, 345, 458–460. 2342578 10.1038/345458a0

[acel14083-bib-0031] Harris, C. R. , Millman, K. J. , van der Walt, S. J. , Gommers, R. , Virtanen, P. , Cournapeau, D. , Wieser, E. , Taylor, J. , Berg, S. , Smith, N. J. , Kern, R. , Picus, M. , Hoyer, S. , van Kerkwijk, M. H. , Brett, M. , Haldane, A. , del Río, J. F. , Wiebe, M. , Peterson, P. , … Oliphant, T. E. (2020). Array programming with NumPy. Nature, 585, 357–362. 32939066 10.1038/s41586-020-2649-2 PMC7759461

[acel14083-bib-0032] Hayflick, L. , & Moorhead, P. S. (1961). The serial cultivation of human diploid cell strains. Experimental Cell Research, 25, 585–621. 13905658 10.1016/0014-4827(61)90192-6

[acel14083-bib-0033] Heckenbach, I. , Mkrtchyan, G. V. , Ezra, M. B. , Bakula, D. , Madsen, J. S. , Nielsen, M. H. , Oró, D. , Osborne, B. , Covarrubias, A. J. , Idda, M. L. , Gorospe, M. , Mortensen, L. , Verdin, E. , Westendorp, R. , & Scheibye‐Knudsen, M. (2022). Nuclear morphology is a deep learning biomarker of cellular senescence. Nature Aging, 2, 742–755. 37118134 10.1038/s43587-022-00263-3 PMC10154217

[acel14083-bib-0034] Hernandez‐Segura, A. , de Jong, T. V. , Melov, S. , Guryev, V. , Campisi, J. , & Demaria, M. (2017). Unmasking transcriptional heterogeneity in senescent cells. Current Biology, 27, 2652–2660. 10.1016/j.cub.2017.07.033 28844647 PMC5788810

[acel14083-bib-0035] Hörl, D. , Rojas Rusak, F. , Preusser, F. , Tillberg, P. , Randel, N. , Chhetri, R. K. , Cardona, A. , Keller, P. J. , Harz, H. , Leonhardt, H. , Treier, M. , & Preibisch, S. (2019). BigStitcher: Reconstructing high‐resolution image datasets of cleared and expanded samples. Nature Methods, 16, 870–874. 31384047 10.1038/s41592-019-0501-0

[acel14083-bib-0036] Hsieh, T.‐H. S. , Cattoglio, C. , Slobodyanyuk, E. , Hansen, A. S. , Rando, O. J. , Tjian, R. , & Darzacq, X. (2020). Resolving the 3D landscape of transcription‐linked mammalian chromatin folding. Molecular Cell, 78, 539–553. 10.1016/j.molcel.2020.03.002 32213323 PMC7703524

[acel14083-bib-1036] Isomura, S. , Wirsching, P. , & Janda, K. D. (2001). An immunotherapeutic program for the treatment of nicotine addiction: Hapten design and synthesis. The Journal of Organic Chemistry, 66(12), 4115–4121. 10.1021/jo001442w 11397142

[acel14083-bib-0037] Ivanov, I. P. , Shin, B. S. , Loughran, G. , Tzani, I. , Young‐Baird, S. K. , Cao, C. , Atkins, J. F. , & Dever, T. E. (2018). Polyamine control of translation elongation regulates start site selection on antizyme inhibitor mRNA via ribosome queuing. Molecular Cell, 70, 254–264. 29677493 10.1016/j.molcel.2018.03.015 PMC5916843

[acel14083-bib-0038] Kang, C. , Xu, Q. , Martin, T. D. , Li, M. Z. , Demaria, M. , Aron, L. , Lu, T. , Yankner, B. A. , Campisi, J. , & Elledge, S. J. (2015). The DNA damage response induces inflammation and senescence by inhibiting autophagy of GATA4. Science, 349, aaa5612. 10.1126/science.aaa5612 26404840 PMC4942138

[acel14083-bib-0039] Kim, Y. H. , Kwak, M. S. , Shin, J. M. , Hayuningtyas, R. A. , Choi, J. E. , & Shin, J. S. (2018). Inflachromene inhibits autophagy through modulation of Beclin 1 activity. Journal of Cell Science, 131, jcs211201. 10.1242/jcs.211201 29361549

[acel14083-bib-0040] Kirschner, K. , Rattanavirotkul, N. , Quince, M. F. , & Chandra, T. (2020). Functional heterogeneity in senescence. Biochemical Society Transactions, 48, 765–773. 32369550 10.1042/BST20190109 PMC7329341

[acel14083-bib-0041] Krietenstein, N. , Abraham, S. , Venev, S. V. , Abdennur, N. , Gibcus, J. , Hsieh, T. H. S. , Parsi, K. M. , Yang, L. , Maehr, R. , Mirny, L. A. , Dekker, J. , & Rando, O. J. (2020). Ultrastructural details of mammalian chromosome architecture. Molecular Cell, 78, 554–565. 32213324 10.1016/j.molcel.2020.03.003 PMC7222625

[acel14083-bib-0042] Krtolica, A. , Parrinello, S. , Lockett, S. , Desprez, P. Y. , & Campisi, J. (2001). Senescent fibroblasts promote epithelial cell growth and tumorigenesis: A link between cancer and aging. Proceedings of the National Academy of Sciences of the United States of America, 98, 12072–12077. 11593017 10.1073/pnas.211053698 PMC59769

[acel14083-bib-0043] Laberge, R. M. , Sun, Y. , Orjalo, A. V. , Patil, C. K. , Freund, A. , Zhou, L. , Curran, S. C. , Davalos, A. R. , Wilson‐Edell, K. A. , Liu, S. , Limbad, C. , Demaria, M. , Li, P. , Hubbard, G. B. , Ikeno, Y. , Javors, M. , Desprez, P. Y. , Benz, C. C. , Kapahi, P. , … Campisi, J. (2015). MTOR regulates the pro‐tumorigenic senescence‐associated secretory phenotype by promoting IL1A translation. Nature Cell Biology, 17, 1049–1061. 26147250 10.1038/ncb3195 PMC4691706

[acel14083-bib-0044] Langmead, B. , & Salzberg, S. L. (2012). Fast gapped‐read alignment with Bowtie 2. Nature Methods, 9, 357–359. 22388286 10.1038/nmeth.1923 PMC3322381

[acel14083-bib-0045] Lee, S. , Nam, Y. , Koo, J. Y. , Lim, D. , Park, J. , Ock, J. , Kim, J. , Suk, K. , & Park, S. B. (2014). A small molecule binding HMGB1 and HMGB2 inhibits microglia‐mediated neuroinflammation. Nature Chemical Biology, 10, 1055–1060. 10.1038/nchembio.1669 25306442

[acel14083-bib-0046] Liao, Y. , Smyth, G. K. , & Shi, W. (2014). featureCounts: An efficient general purpose program for assigning sequence reads to genomic features. Bioinformatics, 30, 923–930. 24227677 10.1093/bioinformatics/btt656

[acel14083-bib-0047] Lopes‐Paciencia, S. , Saint‐Germain, E. , Rowell, M. C. , Ruiz, A. F. , Kalegari, P. , & Ferbeyre, G. (2019). The senescence‐associated secretory phenotype and its regulation. Cytokine, 117, 15–22. 10.1016/j.cyto.2019.01.013 30776684

[acel14083-bib-0048] López‐Otín, C. , Blasco, M. A. , Partridge, L. , Serrano, M. , & Kroemer, G. (2013). The hallmarks of aging. Cell, 153, 1194–1217. 10.1016/j.cell.2013.05.039 23746838 PMC3836174

[acel14083-bib-0049] Love, M. I. , Huber, W. , & Anders, S. (2014). Moderated estimation of fold change and dispersion for RNA‐seq data with DESeq2. Genome Biology, 15, 550. 25516281 10.1186/s13059-014-0550-8 PMC4302049

[acel14083-bib-0050] Lukinavičius, G. , Blaukopf, C. , Pershagen, E. , Schena, A. , Reymond, L. , Derivery, E. , Gonzalez‐Gaitan, M. , D'Este, E. , Hell, S. W. , Gerlich, D. W. , & Johnsson, K. (2015). SiR–Hoechst is a far‐red DNA stain for live‐cell nanoscopy. Nature Communications, 6, 1–7. 10.1038/ncomms9497 PMC4600740 26423723

[acel14083-bib-0051] Madsen, J. G. S. , Schmidt, S. F. , Larsen, B. D. , Loft, A. , Nielsen, R. , & Mandrup, S. (2015). iRNA‐seq: Computational method for genome‐wide assessment of acute transcriptional regulation from total RNA‐seq data. Nucleic Acids Research, 43, e40. 25564527 10.1093/nar/gku1365 PMC4381047

[acel14083-bib-0052] Martin, L. , Schumacher, L. , & Chandra, T. (2023). Modelling the dynamics of senescence spread. Aging Cell, 22, e13892. 10.1111/acel.13892 37288475 PMC10410058

[acel14083-bib-0053] Melnik, S. , Caudron‐Herger, M. , Brant, L. , Carr, I. M. , Rippe, K. , Cook, P. R. , & Papantonis, A. (2016). Isolation of the protein and RNA content of active sites of transcription from mammalian cells. Nature Protocols, 11, 553–565. 26914315 10.1038/nprot.2016.032

[acel14083-bib-0054] Mizi, A. , Zhang, S. , & Papantonis, A. (2020). Genome folding and refolding in differentiation and cellular senescence. Current Opinion in Cell Biology, 67, 56–63. 10.1016/j.ceb.2020.08.002 32911122

[acel14083-bib-0055] Narita, M. , Narita, M. , Krizhanovsky, V. , Nuñez, S. , Chicas, A. , Hearn, S. A. , Myers, M. P. , & Lowe, S. W. (2006). A novel role for high‐mobility group a proteins in cellular senescence and heterochromatin formation. Cell, 126, 503–514. 16901784 10.1016/j.cell.2006.05.052

[acel14083-bib-0056] Narita, M. , Nũnez, S. , Heard, E. , Narita, M. , Lin, A. W. , Hearn, S. A. , Spector, D. L. , Hannon, G. J. , & Lowe, S. W. (2003). Rb‐mediated heterochromatin formation and silencing of E2F target genes during cellular senescence. Cell, 113, 703–716. 12809602 10.1016/s0092-8674(03)00401-x

[acel14083-bib-0057] Oertlin, C. , Lorent, J. , Murie, C. , Furic, L. , Topisirovic, I. , & Larsson, O. (2019). Generally applicable transcriptome‐wide analysis of translation using anota2seq. Nucleic Acids Research, 47, E70. 30926999 10.1093/nar/gkz223 PMC6614820

[acel14083-bib-0058] Olan, I. , Parry, A. J. , Schoenfelder, S. , Narita, M. , Ito, Y. , Chan, A. S. L. , Slater, G. S. C. , Bihary, D. , Bando, M. , Shirahige, K. , Kimura, H. , Samarajiwa, S. A. , Fraser, P. , & Narita, M. (2020). Transcription‐dependent cohesin repositioning rewires chromatin loops in cellular senescence. Nature Communications, 11, 1–14. 10.1038/s41467-020-19878-4 PMC7695716 33247104

[acel14083-bib-0059] Papantonis, A. (2021). HMGs as rheostats of chromosomal structure and cell proliferation. Trends in Genetics, 37, 986–994. 10.1016/j.tig.2021.07.004 34311989

[acel14083-bib-0060] Papaspyropoulos, A. , Hazapis, O. , Altulea, A. , Polizou, K. , Verginis, P. , Evangelou, K. , Fousteri, M. , Papantonis, A. , Gogrgoulis, V. , & Demaria, M. (2023). Decoding of translation‐regulating entities reveals heterogeneous translation deficiency patterns in cellular senescence. Aging Cell, 22, e13893. 10.1111/acel.13893 37547972 PMC10497830

[acel14083-bib-0061] Parry, A. J. , Hoare, M. , Bihary, D. , Hänsel‐Hertsch, R. , Smith, S. , Tomimatsu, K. , Mannion, E. , Smith, A. , D'Santos, P. , Russell, I. A. , Balasubramanian, S. , Kimura, H. , Samarajiwa, S. A. , & Narita, M. (2018). NOTCH‐mediated non‐cell autonomous regulation of chromatin structure during senescence. Nature Communications, 9, 1–15. 10.1038/s41467-018-04283-9 PMC5943456 29743479

[acel14083-bib-0062] Pedregosa, F. , Varoquaux, G. , Gramfort, A. , Michel, V. , Thirion, B. , Grisel, O. , Blondel, M. , Prettenhofer, P. , Weiss, R. , Dubourg, V. , Vanderplas, J. , Passos, A. , Cournapeau, D. , Brucher, M. , Perrot, M. , & Duchesnay, É. (2011). Scikit‐learn: Machine learning in Python. Journal of Machine Learning Research, 12, 2825–2830. 10.5555/1953048.2078195

[acel14083-bib-0063] Rai, T. S. , Cole, J. J. , Nelson, D. M. , Dikovskaya, D. , Faller, W. J. , Vizioli, M. G. , Hewitt, R. N. , Anannya, O. , McBryan, T. , Manoharan, I. , Van Tuyn, J. , Morrice, N. , Pchelintsev, N. A. , Ivanov, A. , Brock, C. , Drotar, M. E. , Nixon, C. , Clark, W. , Sansom, O. J. , … Adams, P. D. (2014). HIRA orchestrates a dynamic chromatin landscape in senescence and is required for suppression of neoplasia. Genes & Development, 28, 2712–2725. 25512559 10.1101/gad.247528.114 PMC4265675

[acel14083-bib-0064] Ramírez, F. , Dündar, F. , Diehl, S. , Grüning, B. A. , & Manke, T. (2014). deepTools: A flexible platform for exploring deep‐sequencing data. Nucleic Acids Research, 42, W187–W191. 24799436 10.1093/nar/gku365 PMC4086134

[acel14083-bib-0065] Risso, D. , Ngai, J. , Speed, T. P. , & Dudoit, S. (2014). Normalization of RNA‐seq data using factor analysis of control genes or samples. Nature Biotechnology, 32, 896–902. 10.1038/nbt.2931 PMC4404308 25150836

[acel14083-bib-0066] Robbins, P. D. , Jurk, D. , Khosla, S. , Kirkland, J. L. , Lebrasseur, N. K. , Miller, J. D. , Passos, J. F. , Pignolo, R. J. , Tchkonia, T. , & Niedernhofer, L. J. (2020). Senolytic drugs: Reducing senescent cell viability to extend health span. Annual Review of Pharmacology and Toxicology, 61, 779–803. 10.1146/annurev-pharmtox-050120-105018 PMC7790861 32997601

[acel14083-bib-0067] Roux, A. E. , Yuan, H. , Podshivalova, K. , Hendrickson, D. , Kerr, R. , Kenyon, C. , & Kelley, D. R. (2022). Individual cell types in *C. elegans* age differently and activate distinct cell‐protective responses. Cell Reports, 42, 112902. 10.1016/j.celrep.2023.112902 37531250

[acel14083-bib-0068] Sadaie, M. , Salama, R. , Carroll, T. , Tomimatsu, K. , Chandra, T. , Young, A. R. J. , Narita, M. , Pérez‐Mancera, P. A. , Bennett, D. C. , Chong, H. , Kimura, H. , & Narita, M. (2013). Redistribution of the Lamin B1 genomic binding profile affects rearrangement of heterochromatic domains and SAHF formation during senescence. Genes & Development, 27, 1800–1808. 23964094 10.1101/gad.217281.113 PMC3759696

[acel14083-bib-0069] Salminen, A. , Ojala, J. , Kaarniranta, K. , & Kauppinen, A. (2012). Mitochondrial dysfunction and oxidative stress activate inflammasomes: Impact on the aging process and age‐related diseases. Cellular and Molecular Life Sciences, 69, 2999–3013. 10.1007/s00018-012-0962-0 22446749 PMC11114788

[acel14083-bib-0070] Sati, S. , Bonev, B. , Szabo, Q. , Jost, D. , Bensadoun, P. , Serra, F. , Loubiere, V. , Papadopoulos, G. L. , Rivera‐Mulia, J. C. , Fritsch, L. , Bouret, P. , Castillo, D. , Gelpi, J. L. , Orozco, M. , Vaillant, C. , Pellestor, F. , Bantignies, F. , Marti‐Renom, M. A. , Gilbert, D. M. , … Cavalli, G. (2020). 4D genome rewiring during oncogene‐induced and replicative senescence. Molecular Cell, 78, 522–538. 10.1016/j.molcel.2020.03.007 32220303 PMC7208559

[acel14083-bib-0071] Satija, R. , Farrell, J. A. , Gennert, D. , Schier, A. F. , & Regev, A. (2015). Spatial reconstruction of single‐cell gene expression data. Nature Biotechnology, 33, 495–502. 10.1038/nbt.3192 PMC4430369 25867923

[acel14083-bib-0072] Schindelin, J. , Arganda‐Carreras, I. , Frise, E. , Kaynig, V. , Longair, M. , Pietzsch, T. , Preibisch, S. , Rueden, C. , Saalfeld, S. , Schmid, B. , Tinevez, J. Y. , White, D. J. , Hartenstein, V. , Eliceiri, K. , Tomancak, P. , & Cardona, A. (2012). Fiji: An open‐source platform for biological‐image analysis. Nature Methods, 9(7), 676–682. 22743772 10.1038/nmeth.2019 PMC3855844

[acel14083-bib-0073] Schmeer, C. , Kretz, A. , Wengerodt, D. , Stojiljkovic, M. , & Witte, O. W. (2019). Dissecting aging and senescence—Current concepts and open lessons. Cell, 8, 1446. 10.3390/cells8111446 PMC6912776 31731770

[acel14083-bib-0074] Sen, P. , Donahue, G. , Li, C. , Egervari, G. , Yang, N. , Lan, Y. , Robertson, N. , Shah, P. P. , Kerkhoven, E. , Schultz, D. C. , Adams, P. D. , & Berger, S. L. (2023). Spurious intragenic transcription is a feature of mammalian cellular senescence and tissue aging. Nature Aging, 3, 402–417. 37117791 10.1038/s43587-023-00384-3 PMC10165726

[acel14083-bib-0075] Shah, P. P. , Donahue, G. , Otte, G. L. , Capell, B. C. , Nelson, D. M. , Cao, K. , Aggarwala, V. , Cruickshanks, H. A. , Rai, T. S. , McBryan, T. , Gregory, B. D. , Adams, P. D. , & Berger, S. L. (2013). Lamin B1 depletion in senescent cells triggers large‐scale changes in gene expression and the chromatin landscape. Genes & Development, 27, 1787–1799. 23934658 10.1101/gad.223834.113 PMC3759695

[acel14083-bib-0076] Sofiadis, K. , Josipovic, N. , Nikolic, M. , Kargapolova, Y. , Übelmesser, N. , Varamogianni‐Mamatsi, V. , Zirkel, A. , Papadionysiou, I. , Loughran, G. , Keane, J. , Michel, A. , Gusmao, E. G. , Becker, C. , Altmüller, J. , Georgomanolis, T. , Mizi, A. , & Papantonis, A. (2021). HMGB1 coordinates SASP‐related chromatin folding and RNA homeostasis on the path to senescence. Molecular Systems Biology, 17, e9760. 34166567 10.15252/msb.20209760 PMC8224457

[acel14083-bib-0077] Sun, L. , Yu, R. , & Dang, W. (2018). Chromatin architectural changes during cellular senescence and aging. Genes, 9, 211. 29659513 10.3390/genes9040211 PMC5924553

[acel14083-bib-0078] Sun, Y. , Coppé, J. P. , & Lam, E. W. F. (2018). Cellular senescence: The sought or the unwanted? Trends in Molecular Medicine, 24, 871–885. 30153969 10.1016/j.molmed.2018.08.002

[acel14083-bib-0079] Teo, Y. V. , Rattanavirotkul, N. , Olova, N. , Salzano, A. , Quintanilla, A. , Tarrats, N. , Kiourtis, C. , Müller, M. , Green, A. R. , Adams, P. D. , Acosta, J. C. , Bird, T. G. , Kirschner, K. , Neretti, N. , & Chandra, T. (2019). Notch signaling mediates secondary senescence. Cell Reports, 27, 997–1007. 10.1016/j.celrep.2019.03.104 31018144 PMC6486482

[acel14083-bib-0080] Van Der Walt, S. , Schönberger, J. L. , Nunez‐Iglesias, J. , Boulogne, F. , Warner, J. D. , Yager, N. , Gouillart, E. , & Yu, T. (2014). Scikit‐image: Image processing in python. PeerJ, 2, e453. 25024921 10.7717/peerj.453 PMC4081273

[acel14083-bib-0081] Vénéreau, E. , Ceriotti, C. , & Bianchi, M. E. (2015). DAMPs from cell death to new life. Frontiers in Immunology, 6, 159317. 10.3389/fimmu.2015.00422 PMC4539554 26347745

[acel14083-bib-0082] Wang, L. , Lankhorst, L. , & Bernards, R. (2022). Exploiting senescence for the treatment of cancer. Nature Reviews. Cancer, 22, 340–355. 35241831 10.1038/s41568-022-00450-9

[acel14083-bib-0083] Wang, W. , Zheng, Y. , Sun, S. , Li, W. , Song, M. , Ji, Q. , Wu, Z. , Liu, Z. , Fan, Y. , Liu, F. , Li, J. , Esteban, C. R. , Wang, S. , Zhou, Q. , Izpisua Belmonte, J. C. , Zhang, W. , Qu, J. , Tang, F. , & Liu, G. H. (2021). A genome‐wide CRISPR‐based screen identifies KAT7 as a driver of cellular senescence. Science Translational Medicine, 13, 2655. 10.1126/scitranslmed.abd2655 33408182

[acel14083-bib-0084] Wiley, C. D. , Flynn, J. M. , Morrissey, C. , Lebofsky, R. , Shuga, J. , Dong, X. , Unger, M. A. , Vijg, J. , Melov, S. , & Campisi, J. (2017). Analysis of individual cells identifies cell‐to‐cell variability following induction of cellular senescence. Aging Cell, 16, 1043–1050. 10.1111/acel.12632 28699239 PMC5595671

[acel14083-bib-0085] Wiley, C. D. , Velarde, M. C. , Lecot, P. , Liu, S. , Sarnoski, E. A. , Freund, A. , Shirakawa, K. , Lim, H. W. , Davis, S. S. , Ramanathan, A. , Gerencser, A. A. , Verdin, E. , & Campisi, J. (2016). Mitochondrial dysfunction induces senescence with a distinct secretory phenotype. Cell Metabolism, 23, 303–314. 10.1016/j.cmet.2015.11.011 26686024 PMC4749409

[acel14083-bib-0086] Zhang, S. , Übelmesser, N. , Barbieri, M. , & Papantonis, A. (2023). Enhancer–promoter contact formation requires RNAPII and antagonizes loop extrusion. Nature Genetics, 55, 832–840. 37012454 10.1038/s41588-023-01364-4

[acel14083-bib-0087] Zhang, X. , Liu, X. , Du, Z. , Wei, L. , Fang, H. , Dong, Q. , Niu, J. , Li, Y. , Gao, J. , Zhang, M. Q. , Xie, W. , & Wang, X. (2021). The loss of heterochromatin is associated with multiscale three‐dimensional genome reorganization and aberrant transcription during cellular senescence. Genome Research, 31, 1121–1135. 34140314 10.1101/gr.275235.121 PMC8256869

[acel14083-bib-0088] Zhou, Y. , Zhou, B. , Pache, L. , Chang, M. , Khodabakhshi, A. H. , Tanaseichuk, O. , Benner, C. , & Chanda, S. K. (2019). Metascape provides a biologist‐oriented resource for the analysis of systems‐level datasets. Nature Communications, 10, 1523. 10.1038/s41467-019-09234-6 PMC6447622 30944313

[acel14083-bib-0089] Zirkel, A. , Nikolic, M. , Sofiadis, K. , Mallm, J. P. , Brackley, C. A. , Gothe, H. , Drechsel, O. , Becker, C. , Altmüller, J. , Josipovic, N. , Georgomanolis, T. , Brant, L. , Franzen, J. , Koker, M. , Gusmao, E. G. , Costa, I. G. , Ullrich, R. T. , Wagner, W. , Roukos, V. , … Papantonis, A. (2018). HMGB2 loss upon senescence entry disrupts genomic organization and induces CTCF clustering across cell types. Molecular Cell, 70, 730–744. 10.1016/j.molcel.2018.03.030 29706538

