# Supplementary material for: Rapid and synchronous chemical induction of replicative‐like senescence via a small molecule inhibitor
Source: Aging Cell. 2024 Jan 9;23(4):e14083. doi: 10.1111/acel.14083 (PMC11019153; doi:10.1111/acel.14083)
Supplement: Supplementary file 1 — Appendix S1 [file ACEL-23-e14083-s001.zip › acel14083-sup-0001-Supinfo.pdf]

SUPPORTING INFORMATION

Rapid and synchronous chemical induction of replicative-like senescence via a small molecule inhibitor

Spiros Palikyras, *et al*.

Contents

This sections contains supplementary **Figures S1-S8** and **Tables S1-S7**.

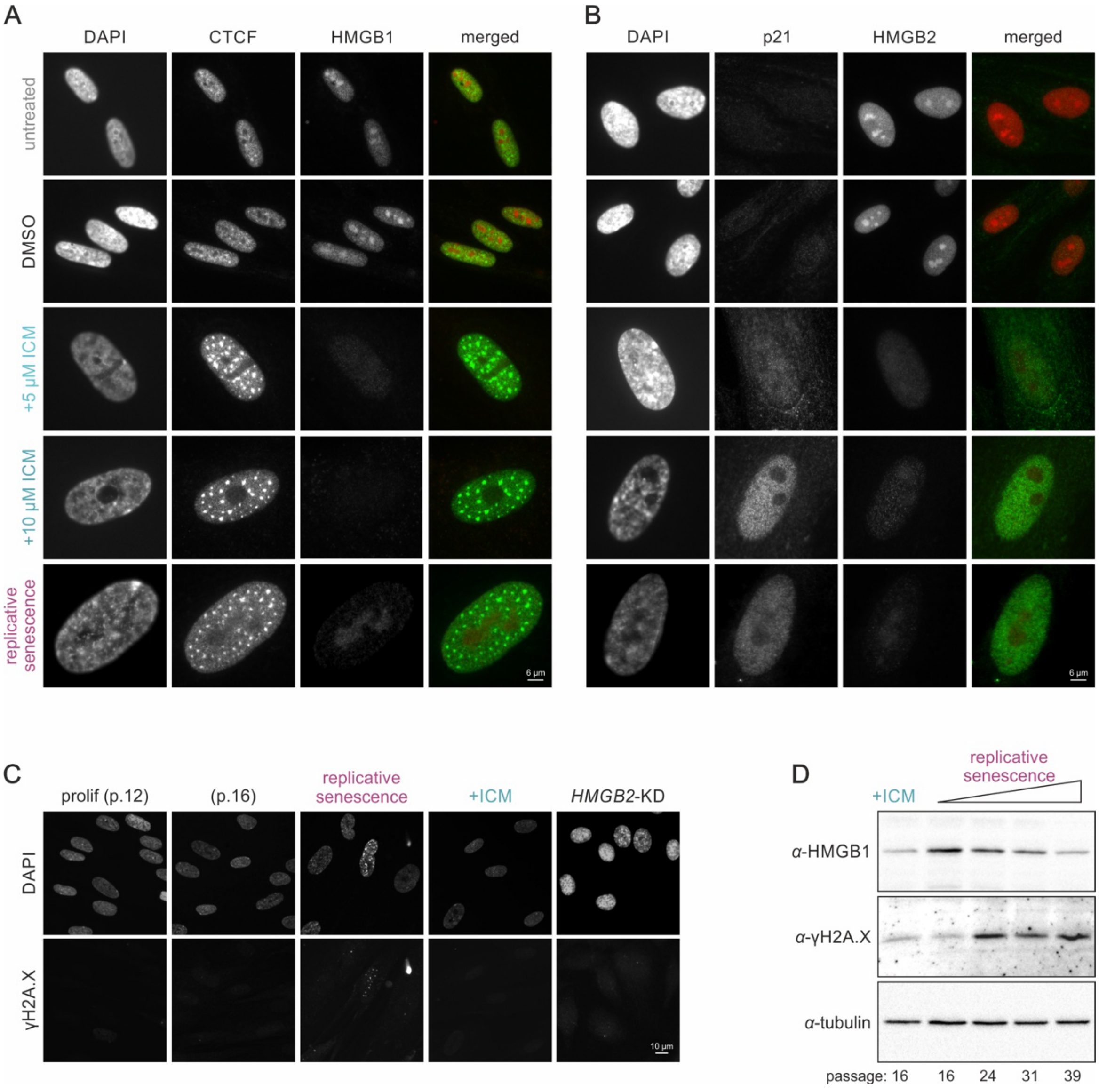

FIGURE S1 ICM treatment depletes HMGB1 and -B2 from IMR90 cell nuclei.
(**A**) Representative widefield images of proliferating, DMSO-, and 3- or 6-day ICM-treated IMR90 immunostained for CTCF and HMGB1, and counterstained with DAPI. Bar: 6 µm. (**B**) As in panel A, but immunostained for p21 and HMGB2. Bar: 6 µm. (**C**) As in panel A, but immunostained for phospho-γ-H2A.X. (**D**) Western blots showing changing HMGB1 and phospho-γ-H2A.X levels in ICM-treated or progressively passaged IMR90. Tubulin levels provide a loading control.

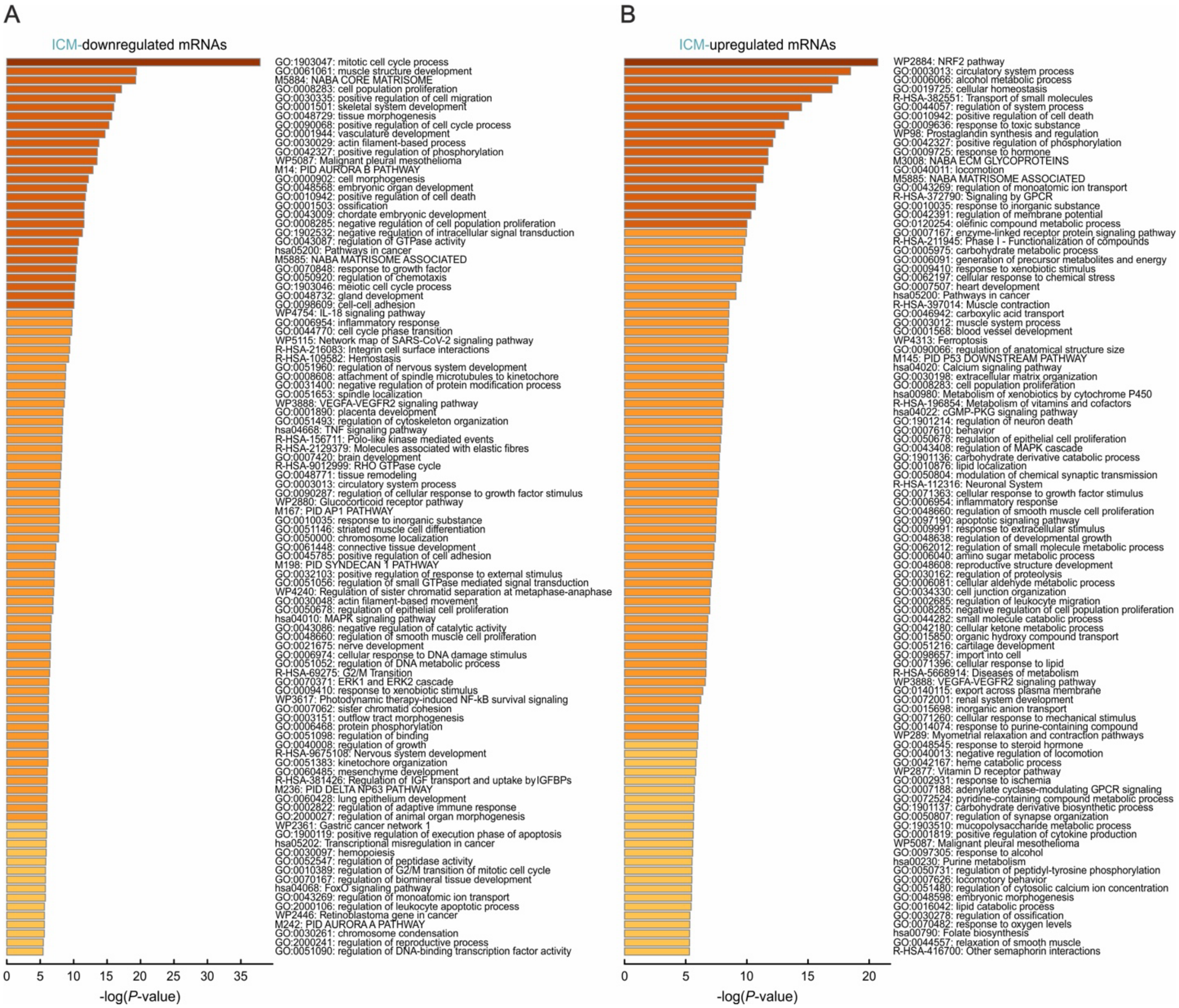

FIGURE S2 Effects of ICM treatment at the level of mRNA in IMR90.
(**A**) Top 100 GO terms/pathways associated with mRNAs downregulated following 6 days of 10 µM ICM treatment.
(**B**) As in panel A, but for ICM-upregulated mRNAs.

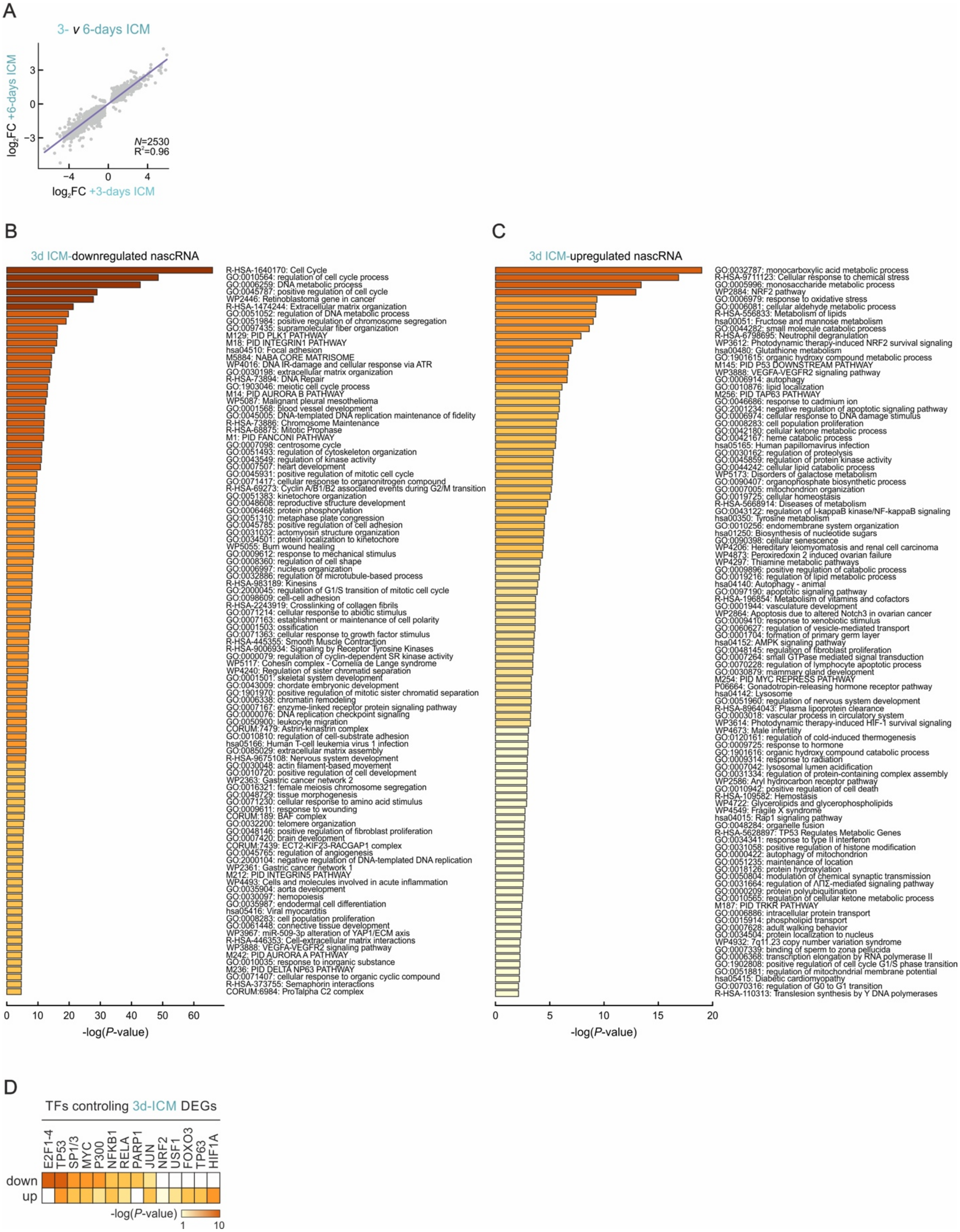

FIGURE S3 Effects of 3-day ICM treatment at the level of nascent RNA production in IMR90.
(**A**) Plot correlating changes ($\log_2FC$) in nascent RNA levels of genes regulated after 3 and 6 days of ICM treatment. The number of the queried genes (*N*) and the deduced Spearman's correlation coefficient (R^2) are shown. (**B**) Top 100 GO

terms/pathways associated with nascent RNAs downregulated after 3 days of 10 μM ICM treatment. (**C**) As in panel B, but for 3-day ICM-upregulated nascent RNAs. (**D**) Heatmap showing transcription factors (TFs) predicted to bind ICM-regulated genes from panels A and B based on TTRUST motif enrichment.

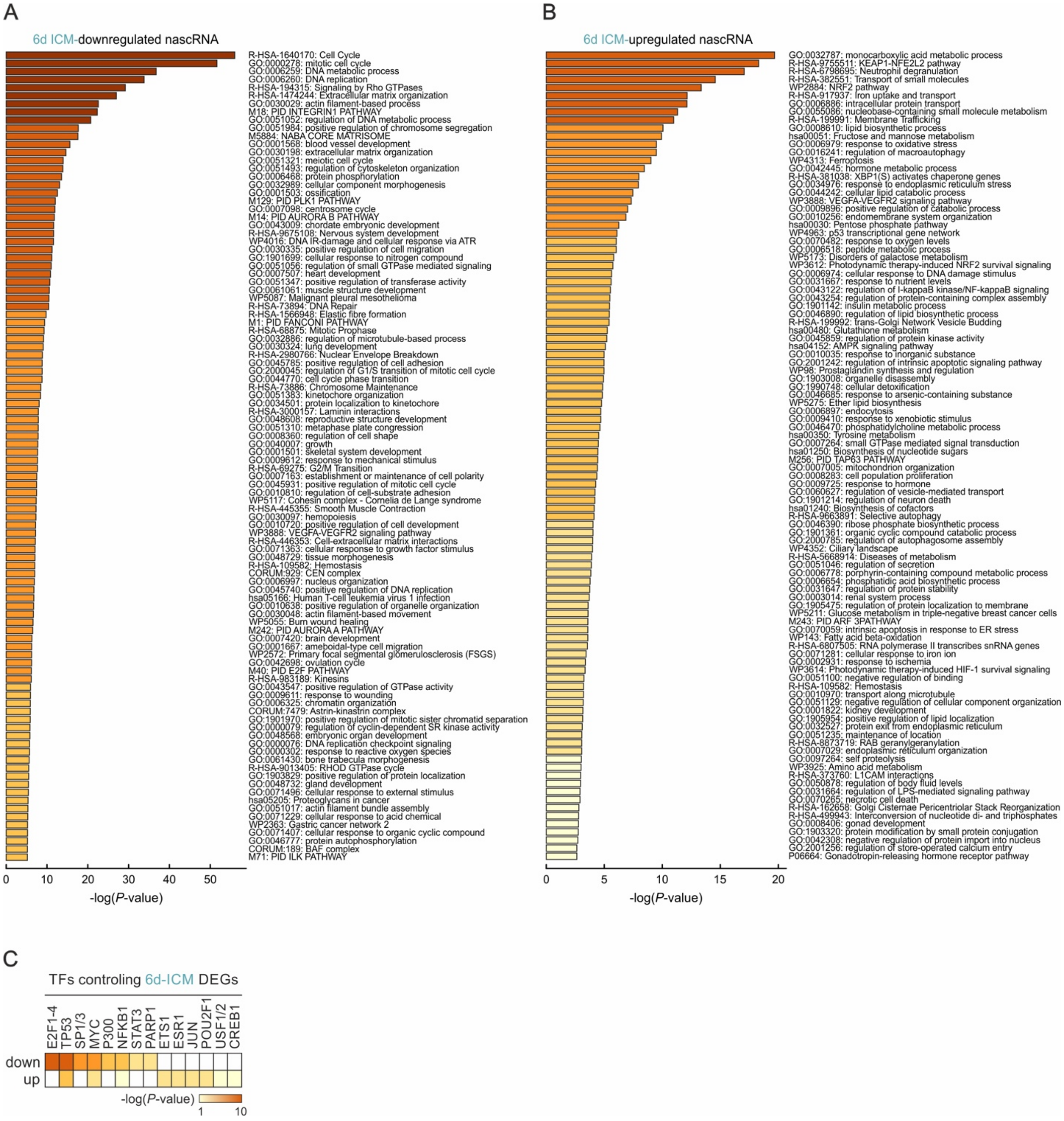

FIGURE S4 Effects of 6-dayICM treatment at the level of nascent RNA production in IMR90.
(**A**) Top 100 GO terms/pathways associated with nascent RNAs downregulated after 6 days of 10 µM ICM treatment. (**B**) As in panel A, but for 6-day ICM-upregulated nascent RNAs. (**C**) Heatmap showing transcription factors (TFs) predicted to bind ICM-regulated genes from panels A and B based on TTRUST motif enrichment.

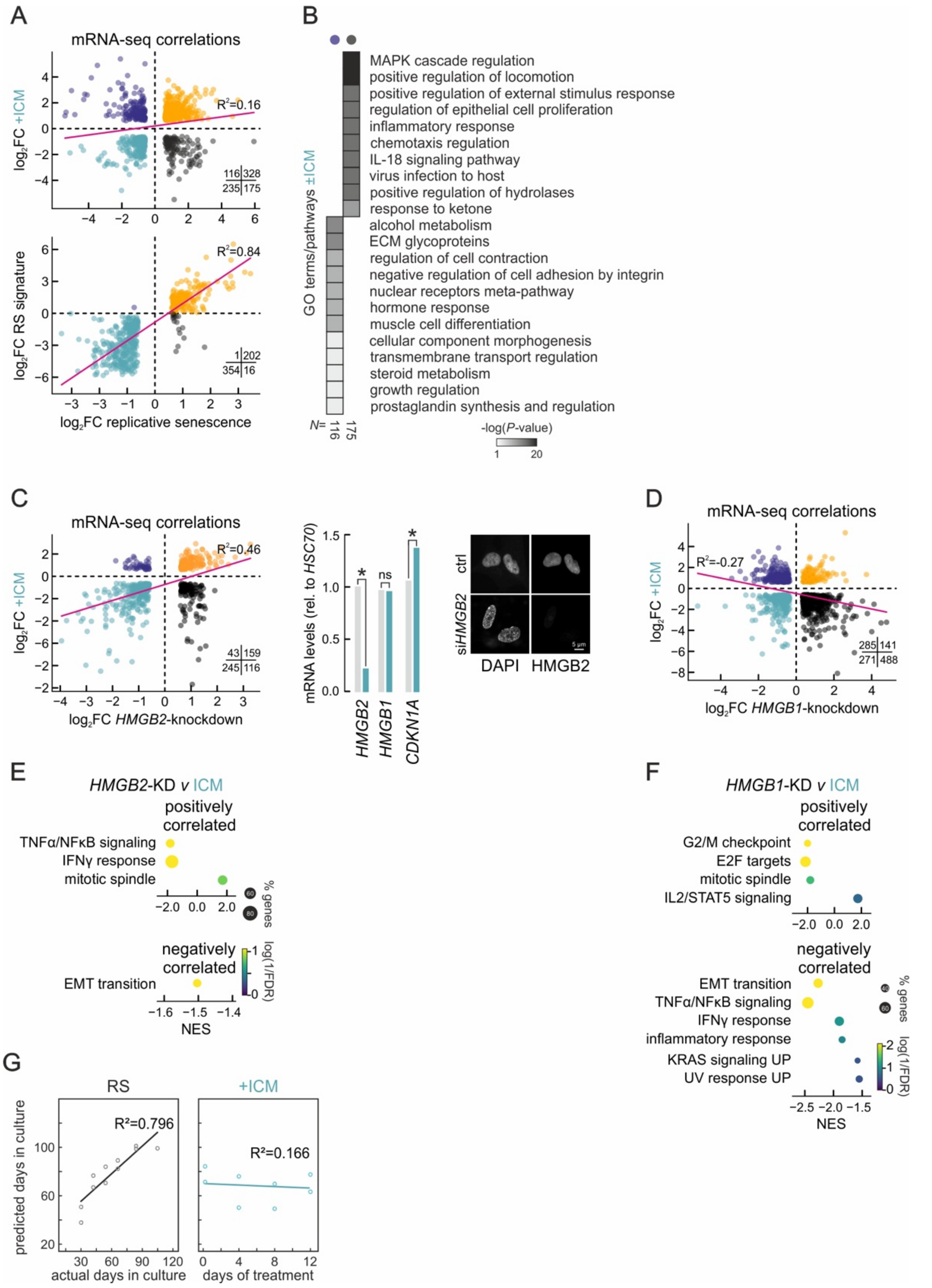

FIGURE S5 Transcriptional changes and methylation clock in 6-day ICM-treated IMR90.

(**A**) Top: Scatter plots showing correlation of differentially expressed mRNAs from ICM-treated and replicatively senescent IMR90. Bottom: As above, but for mRNAs from ICM-treated IMR90 and a consensus senescence signature. Spearman's correlation coefficients (R^2) and the number of genes in each quadrant (*N)* are shown. (**B**) Heatmap of GO terms/pathways associated with the two diverging gene subsets from panel A (up in ICM and down in RS – purple; down in ICM and up in RS – black). The number of genes in each subset (*N*) is indicated. (**C**) Left: As in panel A, but correlating differentially expressed mRNAs from ICM-treated and *HMGB2*-knockdown IMR90. Middle: Bar plot

showing relative mRNA levels (mean of two RT-qPCR replicates) of *HMGB2*, *-B1*, and *CDKN1A* upon 72 h of *HMGB2* knockdown. **P*<0.05, unpaired two-tailed Student's t-test. Right: Representative IMR90 immunofluorescence imaging showing HMGB2 nuclear depletion upon 72 h of *HMGB2* knockdown. Bar: 5 μM. (**D**) As in panel A, but correlating differentially expressed mRNAs from ICM-treated and *HMGB1*-knockdown IMR90. (**E**) Plots showing GSEA results for mRNAs correlating positively (green/orange data points) or negatively (blue/black data points) in panel C. (**F**) As in panel E, but using positively/negatively correlated mRNAs from panel D. (**G**) Plots correlating IMR90 passage predicted by methylation changes at six senescence-associated CpGs with actual passage (left) or days of ICM treatment (right). Spearman's correlation coefficients (R^2) are shown.

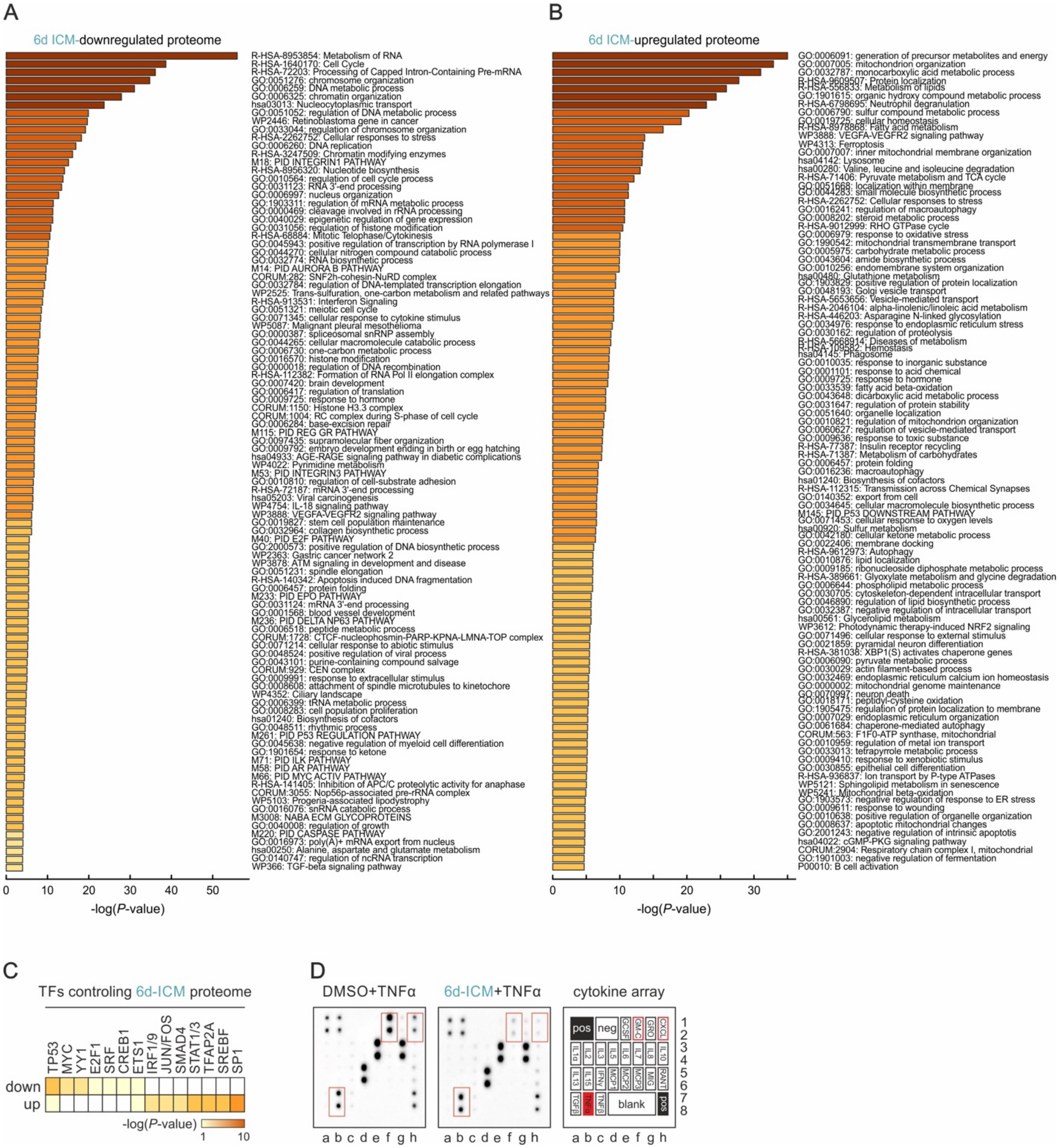

FIGURE S6 Effects of ICM treatment at the level of IMR90 whole-cell proteome and secretome.

(**A**) Top 100 GO terms/pathways associated with proteins downregulated after 6 days of 10 µM ICM treatment. (**B**) As in panel A, but for ICM-upregulated proteins. (**C**) Heatmap showing transcription factors (TFs) predicted to bind the genes encoding the ICM-regulated genes from panels A and B based on TTRUST motif enrichment. (**D**) Cytokine profiling of the supernatant from TNFα-treated proliferating (DMSO) or ICM-induced (for 6 days) IMR90 cultures.

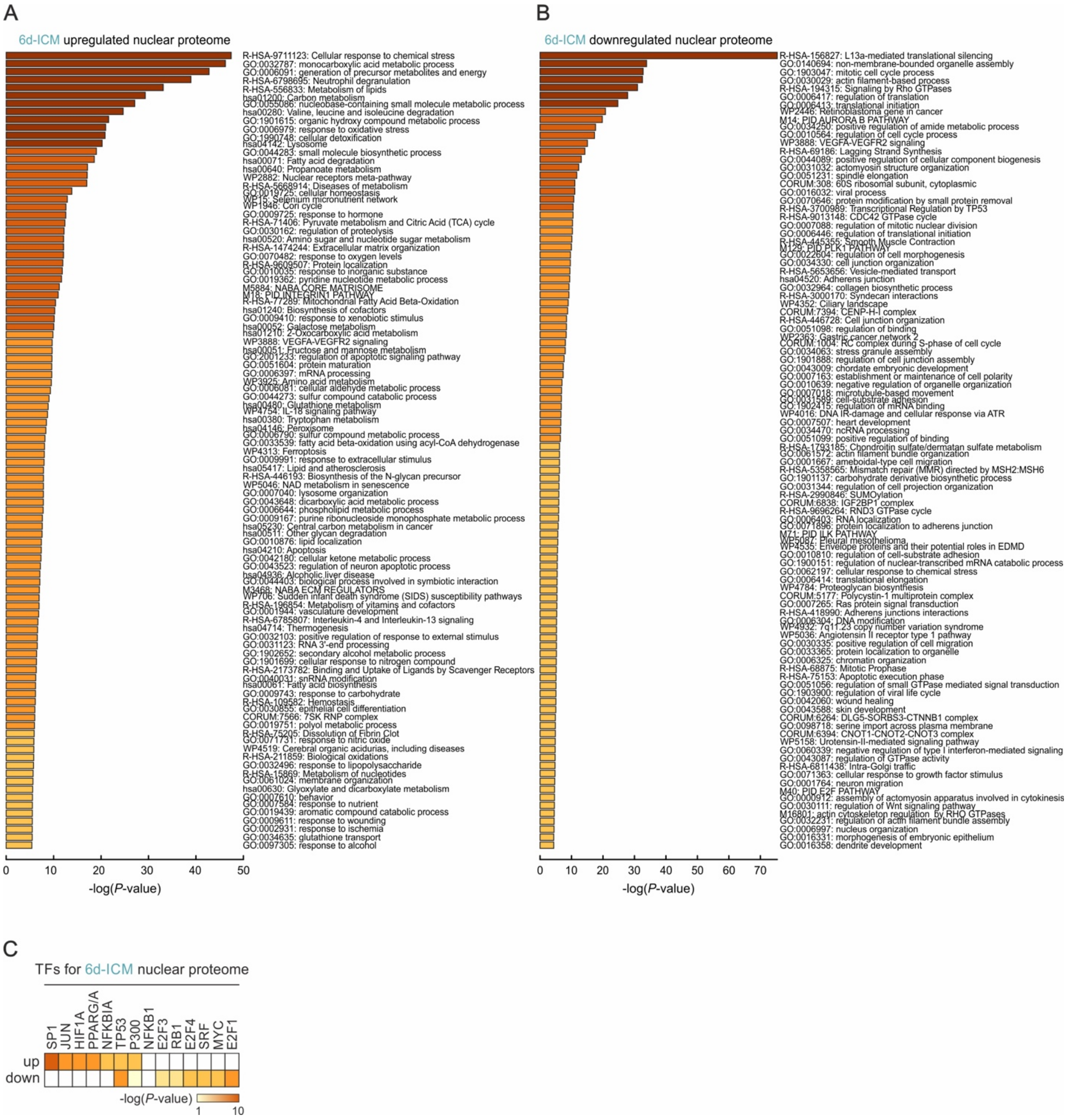

FIGURE S7 Effects of ICM treatment at the level of IMR90 nuclear proteome.
(**A**) Top 100 GO terms/pathways associated with nuclear proteins upregulated after 6 days of 10 µM ICM treatment. (**B**) As in panel A, but for ICM-downregulated nuclear proteins. (**C**) Heatmap showing TFs predicted to bind the genes encoding the ICM-regulated genes from panels A and B based on TTRUST motif enrichment.

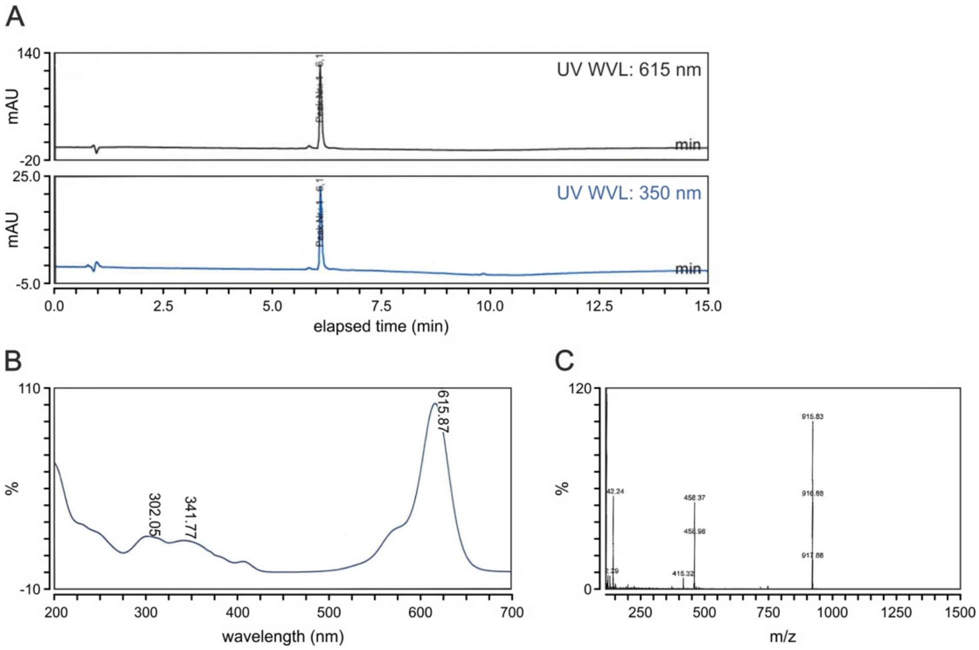

FIGURE S8 Characterization of ICM-C6-610CP.
(**A**) Analytical LC trace showing high purity of the ICM-C6-610CP compound. (**B**) Absorbance spectrum of ICM-C6-610CP recorded at 6.1-min retention time of analytical LC. (**C**) Mass spectrum of ICM-C6-610CP theoretical m/z corresponding to 915.44.

Table S1. Basic mapping statistics of Micro-C experiments.

	proliferating (DMSO-treated)	ICM-treated (6 days)
total reads	1,175,415,686	1,238,470,006
% mapped	82	81
% duplicates	30	23
total valid	614,188,035	711,139,837
total *cis*	417,456,327	488,155,052
total *trans*	83,549,563	89,834,674
% long range	46	52

Table S2. List of primers used in RT-qPCR experiments.

gene target	forward primer (5'-3')	reverse priimer (5'-3')
HSC70	TTATTGGAGCCAGGCCTACAC	GCGACATAGCTTGGAGTGGT
LMNB1	CTGGCCAAGATGTGAAGGTTA	TCCTCTTCTTCAGGTATGGTTGTT
HMGB1	TGAGCTCCATAGAGACGCG	GATGACATTTTGCCTCTCGG
HMGB2	CCAATGCTCCTAAAAGGCCACC	CCAATGGATAGGCCAGGGTGTT
HMGA1	GAAAAGGACGGCACTGAGAA	CCCCGAGGTCTCTTAGGTGT
HDAC9	CATGAGAACTTGACACGGCA	TGCTCCAGTTTCTGCTCCTT
CCND2	TGGCCTCCAAACTCAAAGAG	CACTTCAACTTCCCCAGCAC
CDKN1A	TGGAGACTCTCAGGGTCGAA	GGATTAGGGCTTCCTCTTGG
SMC1	GGGGAGAAGACAGTGGCAG	TTGGTGTTATCCAAGGCAGC
CTCF	CAGAGGTTAATGCAGAGAAAGTG	AATGCCATGCCACAGAGATG
RBL1	GTATTCCAAGAGAAGTTGTGGCA	GGTCCACTGGAACAGTCAGG

Table S3. List of differentially expressed genes identified by RNA-seq approaches.
(Provided as an *.xlsx* file.)

Table S4. List of primers used in HMGB2 ChIP-qPCR experiments.

pair	forward primer (5'-3')	reverse primer (5'-3')	location (hg19)
#a	ATGCGGGTTTACCATGCAGA	GGCCGAGAGCCATAAAGACA	chr10:92,671,441-92,671,590
#b	TCAGACTCCGCAGGAAAGGT	AAGTACGCGCCTTGGTGAG	chr1:205,091,401-205,091,550
#c	CATACAATAAAGGTGGTGCCAG	TTCAGGAGCTTAATACTGGAGGC	chr15: 39,890,983-39,891,451
#d	GACACGCTCAATAGGCTGAGT	GGGCTCTTATCCTTTCCCGA	chr20: 43,229,526-43,229,628
#e	TGAGTGCCATTCACTTAACAGC	GCTGGTAGTGGCTACCTTACG	chr10: 12,085,032-12,085,261
#f	TCAGTGCGAAGCCGATTTCC	AACATCTTTCGACTCCGCCC	chr3:197,676,616-197,677,150

Table S5. List of differentially translated/buffered mRNAs identified by Ribo-seq experiments.
(Provided as an *.xlsx* file.)

Table S6. List of peptides identified by whole-cell proteomics and associated statistics.
(Provided as an *.xlsx* file.)

Table S7. List of peptides identified by proteomics on fractionated IMR90 and associated statistics.
(Provided as an *.xlsx* file.)
